# Genetic Evidence for Unified Airway Disease: Shared Epithelial and Immune Architecture Across Major Airway Diseases

**DOI:** 10.3390/ijms27167450

**Published:** 2026-08-20

**Authors:** Tianqi Tu, Yongjin Guo, Qing Li, Yutong Liu, Liying Jiang

**Affiliations:** 1 School of Public Health, Shanghai University of Traditional Chinese Medicine, Shanghai 201203, China; 2 School of Public Health, Shanghai University of Medicine & Health Sciences, Shanghai 201318, China; 3 Center for Excellence in Brain Science and Intelligence Technology, Institute of Neuroscience, Chinese Academy of Sciences, Shanghai 200031, China

**Keywords:** Genomic SEM, COPD, asthma, bronchiectasis, chronic rhinosinusitis

## Abstract

Major airway diseases, including chronic obstructive pulmonary disease (COPD), asthma, bronchiectasis and chronic rhinosinusitis without nasal polyps (CRSsNP), frequently coexist and share inflammatory, epithelial and remodeling features. However, whether these clinically distinct airway disorders are driven by a unified genetic liability and how this shared liability maps to disease-relevant tissues, genes and immune-regulatory programs remain incompletely understood. We integrated GWAS summary statistics for COPD, asthma, bronchiectasis and CRSsNP using linkage disequilibrium score regression, local genetic correlation analysis and Genomic structural equation modeling. A latent shared airway disease factor, termed gAirwayDisease, was constructed to capture common genetic liability across the four conditions. We then applied an integrative functional genomics framework, including gsMap spatial enrichment, PoPS gene prioritization, MAGMA gene-set enrichment, GTEx v8 lung MTWAS, OneK1K and DICE immune-cell MTWAS, scMORE regulon analysis and phenome-wide Mendelian randomization. All six airway disease pairs showed positive genetic correlations, with estimates ranging from 0.508 to 0.685. Genomic SEM supported a single shared factor, with positive standardized loadings for COPD, asthma, bronchiectasis and CRSsNP and excellent model fit. Spatial mapping localized gAirwayDisease-associated signals to airway- and epithelial-associated anatomical domains. PoPS prioritized immune and airway-relevant genes, including SMAD3, GATA3, IL1R1, RUNX3 and STAT6, while MAGMA enrichment highlighted B-cell activation, T-cell activation and transcriptional regulatory pathways. Lung MTWAS identified SLC9A2 and ORMDL3 as top genetically regulated expression signals. OneK1K immune-cell MTWAS highlighted recurrent IL18R1 associations across CD4 and CD8 T-cell subsets. scMORE further identified 36 significant regulon–cell type pairs across dendritic cells, B cells, monocytes, T cells and NK cells, including BCL11A, TCF4, KLF4, RUNX1 and STAT4 regulons. MR-PheWAS linked genetically predicted gAirwayDisease to respiratory, allergic, lung function and immune-related traits. This study defines gAirwayDisease as a genetically informed latent factor capturing shared liability across major airway diseases. Integrated functional genomic analyses highlight airway epithelial and immune regulatory programs associated with shared disease susceptibility and prioritize candidate genes and regulons for future experimental validation.

## 1. Introduction

Major airway diseases, including chronic obstructive pulmonary disease (COPD), asthma, bronchiectasis, and chronic rhinosinusitis without nasal polyps (CRSsNP), impose substantial global health burdens and frequently coexist within individuals [1,2]. Although these conditions differ in anatomical distribution, clinical presentation, and disease course, they share key pathological features, including chronic airway inflammation, epithelial barrier dysfunction, mucus dysregulation, and airway remodeling [1,3]. These four disorders were selected because they represent chronic airway diseases spanning the upper and lower respiratory tract, frequently coexist, and share pathological features including chronic inflammation, epithelial dysfunction, mucus dysregulation, and airway remodeling [1,2,3]. CRSsNP was included as a non-polypoid upper-airway phenotype to determine whether shared genetic liability extends across anatomically distinct airway compartments. The present analysis was not intended to encompass all chronic airway diseases. Other conditions were not included when they were predominantly monogenic or etiologically distinct, substantially overlapped with the selected phenotypes, or lacked sufficiently powered and harmonized GWAS summary statistics suitable for Genomic SEM. Genome-wide association studies (GWAS) have identified numerous susceptibility loci for individual airway diseases [4,5]. However, most studies have examined these disorders separately, leaving unresolved whether clinically distinct airway diseases are partly driven by a common genetic liability.

Genetic correlation studies have reported shared genetic signal between COPD and allergic diseases, and between asthma and chronic rhinosinusitis, supporting the concept of a genetically connected airway disease spectrum [2,6]. However, pairwise genetic correlations quantify overlap between trait pairs but do not define a unified liability factor across multiple airway diseases, nor do they identify the tissues, genes, cell types, or regulatory programs associated with shared genetic risk [7]. Genomic structural equation modeling (Genomic SEM) addresses this limitation by modeling the genetic covariance structure among complex traits and deriving latent factors that capture shared genetic architecture [7]. This approach has been applied to psychiatric and other complex diseases to identify transdiagnostic genetic factors, detect loci not evident in single-trait GWAS, and clarify shared biological pathways [7,8].

A central challenge in post-GWAS interpretation is linking associated variants to disease-relevant tissues, cell types, and effector genes [4,9]. Integrative approaches that combine GWAS with expression quantitative trait loci, gene-prioritization frameworks, and cell-type-specific regulatory resources can help bridge this gap [9,10]. In airway disease genetics, lung and airway eQTL analyses have nominated candidate causal genes at COPD and asthma risk loci, while cell-type-aware studies have revealed regulatory effects that may be masked in bulk tissue analyses [11,12]. Because immune dysregulation is central to airway disease pathogenesis, resolving the immune-cell types and transcriptional programs mediating shared genetic risk remains an important unmet need [13]. Single-cell eQTL and chromatin accessibility studies further show that genetic effects on gene regulation are often highly cell-type-specific and context-dependent across immune subsets and disease states [12,14,15].

In this study, we applied Genomic SEM to GWAS summary statistics for COPD, asthma, bronchiectasis, and CRSsNP to define a latent shared airway disease factor, termed gAirwayDisease. We then used an integrative functional genomics framework to map gAirwayDisease to anatomical regions, candidate effector genes, lung tissue expression, immune-cell-specific expression, and transcription factor regulons. This study aimed to characterize the shared genetic architecture of major airway diseases and identify epithelial and immune regulatory programs associated with common airway disease liability.

## 2. Results

### 2.1. Shared SNP Heritability and Genetic Correlation Across Airway Diseases

**Figure 1:**
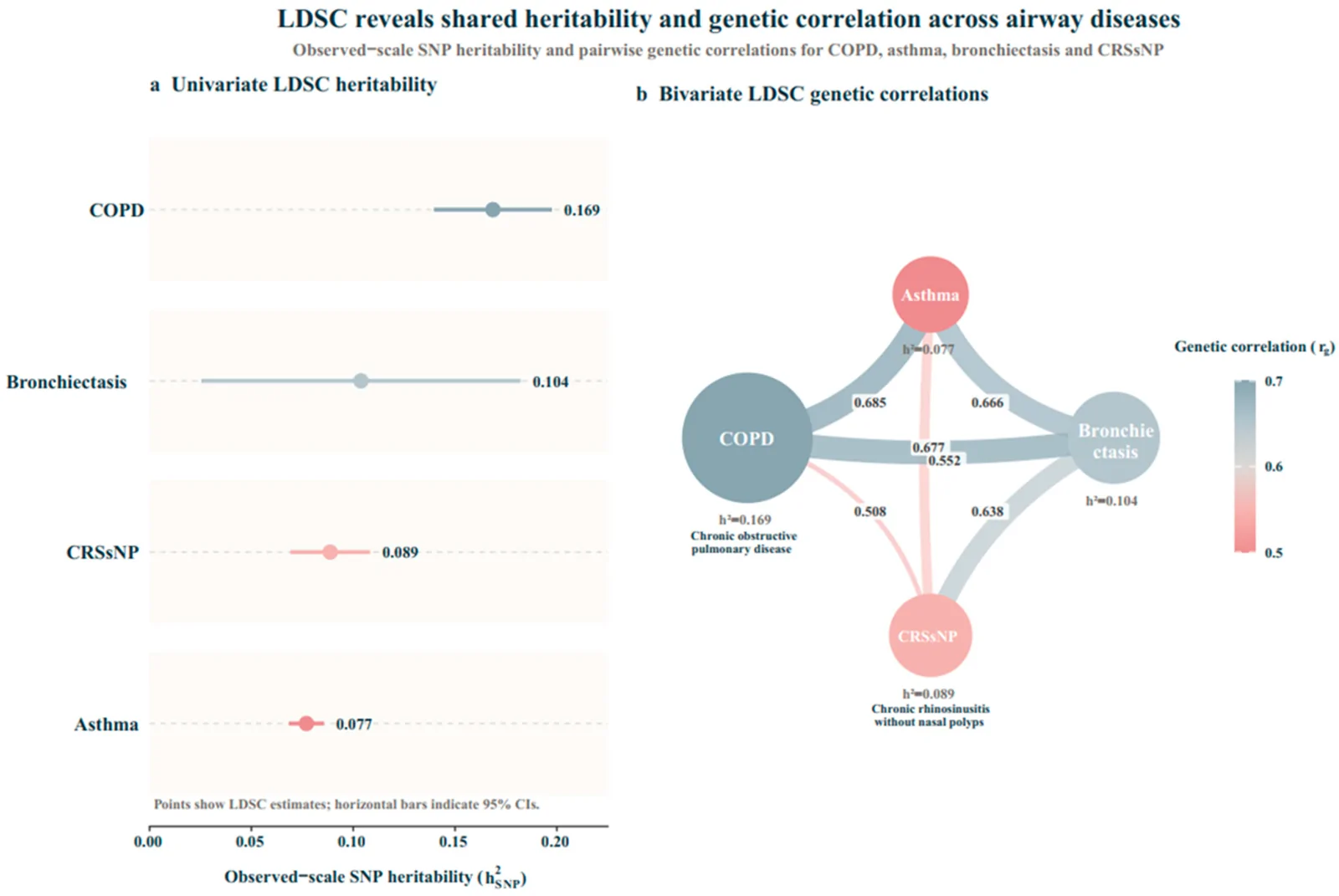
Shared SNP heritability and genetic correlation across airway diseases revealed by LDSC. (**a**) Observed-scale SNP heritability estimated by univariate LDSC for four airway diseases, including COPD, asthma, bronchiectasis and CRSsNP. Points represent LDSC heritability estimates and horizontal bars indicate 95% confidence intervals. COPD showed the highest SNP heritability estimate (h^2^_SNP_ = 0.169), followed by bronchiectasis (h^2^_SNP_ = 0.104), CRSsNP (h^2^_SNP_ = 0.089) and asthma (h^2^_SNP_ = 0.077). (**b**) Pairwise LDSC genetic correlation network among the four airway diseases. Node size is proportional to the observed-scale SNP heritability estimate, and edge width and color intensity represent the magnitude of the genetic correlation (rg). All pairwise genetic correlations were positive and Bonferroni-significant, indicating a shared polygenic architecture across airway diseases. The strongest correlations were observed between COPD and asthma (rg = 0.685), COPD and bronchiectasis (rg = 0.677), and asthma and bronchiectasis (rg = 0.666), with additional positive correlations for bronchiectasis–CRSsNP (rg = 0.638), asthma–CRSsNP (rg = 0.552) and COPD–CRSsNP (rg = 0.508). Abbreviations: COPD, chronic obstructive pulmonary disease; CRSsNP, chronic rhinosinusitis without nasal polyps; LDSC, linkage disequilibrium score regression; h^2^_SNP_, SNP-based heritability; rg, genetic correlation.

We first assessed the genome-wide genetic architecture of four major airway diseases: COPD, asthma, bronchiectasis, and CRSsNP. Univariate LDSC showed detectable observed-scale SNP-based heritability for all four traits. COPD had the highest estimate (h^2^SNP = 0.169), followed by bronchiectasis (h^2^SNP = 0.104), CRSsNP (h^2^SNP = 0.089), and asthma (h^2^SNP = 0.077) (Figure 1a). LDSC intercepts were close to 1.0 across datasets, indicating that inflation in GWAS test statistics was largely attributable to polygenic signal rather than confounding. Detailed GWAS sources, sample sizes, effective sample sizes, heritability estimates, and LDSC inflation metrics are provided in Table S1.

Bivariate LDSC revealed positive genetic correlations across all six airway disease pairs (Figure 1b). The strongest correlations were observed for COPD–asthma (rg = 0.685), COPD–bronchiectasis (rg = 0.677), and asthma–bronchiectasis (rg = 0.666). Additional positive correlations were observed for bronchiectasis–CRSsNP (rg = 0.638), asthma–CRSsNP (rg = 0.552), and COPD–CRSsNP (rg = 0.508). All pairwise genetic correlations remained significant after Bonferroni correction. Full genetic covariance and correlation estimates are provided in Table S2.

### 2.2. Local Genetic Correlation Reveals Region-Specific Sharing Across Airway Disease Pairs

**Figure 2:**
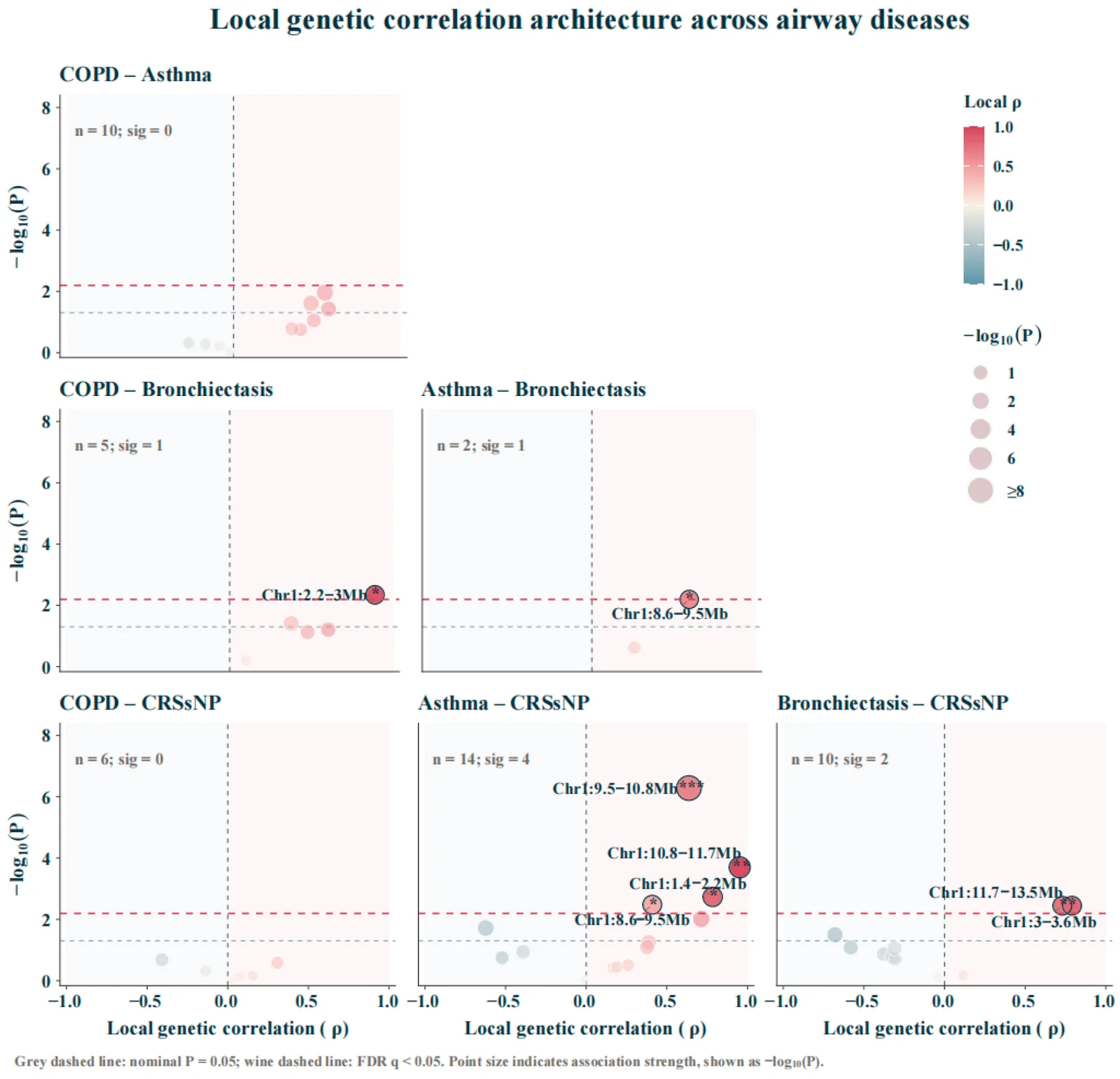
Local genetic correlation architecture across airway diseases. Pairwise local genetic correlations among chronic obstructive pulmonary disease (COPD), asthma, bronchiectasis and chronic rhinosinusitis without nasal polyps (CRSsNP) were estimated using LAVA. Each volcano panel represents one disease pair and summarizes locus-level bivariate local genetic correlation signals across semi-independent genomic regions. The x axis shows the estimated local genetic correlation coefficient (ρ), and the y axis shows −log_10_(*p*) from the LAVA bivariate test. Points represent genomic loci. The blue-to-red gradient denotes the direction and magnitude of local genetic correlation, with blue indicating negative correlation, ivory indicating near-zero correlation and wine red indicating positive correlation. Point size reflects statistical evidence, measured by −log_10_(*p*). Outlined points indicate loci reaching the selected significance threshold based on FDR *q* < 0.05. The gray dashed horizontal line denotes nominal *p* = 0.05, and the wine dashed line denotes the FDR-based significance threshold. Labeled loci represent the strongest significant local genetic correlation signals within each disease pair. FDR values were calculated from the LAVA bivariate *p* values using the Benjamini–Hochberg procedure. Abbreviations: COPD, chronic obstructive pulmonary disease; CRSsNP, chronic rhinosinusitis without nasal polyps; FDR, false discovery rate; local rg, local genetic correlation. Significance levels are indicated as follows: * *q* < 0.05, ** *q* < 0.01, and *** *q* < 0.001.

LAVA identified eight FDR-significant local genetic correlation signals across the six airway disease pairs, all showing positive local sharing (Figure 2). The most prominent signals involved asthma–CRSsNP, with four significant chr1 loci, including the strongest signal at chr1:9.5–10.8 Mb (ρ = 0.637, *p* = 5.20 × 10^−7^, FDR q = 2.44 × 10^−5^). Additional positive local sharing was observed for bronchiectasis–CRSsNP at chr1:11.7–13.5 Mb and chr1:3.0–3.6 Mb, for COPD–bronchiectasis at chr1:2.2–3.0 Mb, and for asthma–bronchiectasis at chr1:8.6–9.5 Mb. These findings extend the global LDSC results by showing that shared airway disease genetics includes both genome-wide and locus-specific components.

### 2.3. Genomic SEM Defines gAirwayDisease as a Latent Shared Airway Disease Factor

**Figure 3:**
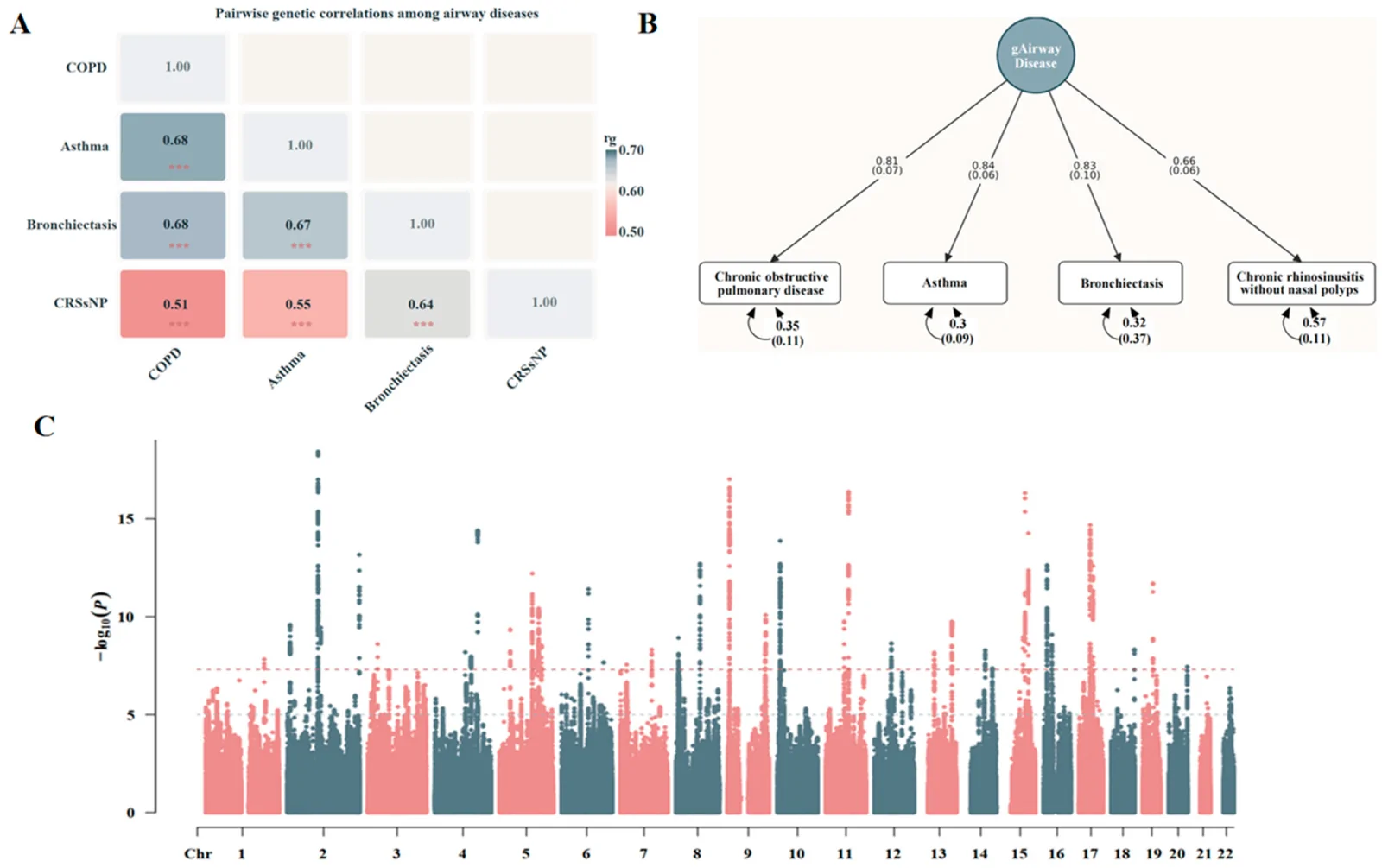
Genomic SEM identifies a shared genetic architecture underlying major airway diseases. (**A**) Pairwise genetic correlations among four airway diseases, including chronic obstructive pulmonary disease (COPD), asthma, bronchiectasis and chronic rhinosinusitis without nasal polyps (CRSsNP). Values within cells indicate genetic correlation estimates (rg). The diagonal represents self-correlations. Color intensity reflects the magnitude of genetic correlation, with red indicating lower correlations and blue indicating higher correlations. Asterisks denote statistical significance, with *** indicating *p* < 0.001. (**B**) Genomic structural equation model defining a latent airway disease factor, gAirwayDisease, from the shared genetic covariance among COPD, asthma, bronchiectasis and CRSsNP. Standardized factor loadings are shown on the paths from gAirwayDisease to each observed disease, with standard errors in parentheses. Residual genetic variances for each disease are shown beneath the corresponding phenotype box. All four diseases loaded positively onto the common factor, with factor loadings of 0.81 for COPD, 0.84 for asthma, 0.83 for bronchiectasis and 0.66 for CRSsNP, supporting a shared genetic liability across airway diseases. (**C**) Manhattan plot of the genome-wide association analysis for the GSEM-derived gAirwayDisease factor. Each point represents a genetic variant plotted according to chromosomal position and association strength, shown as −log_10_(*p*). Alternating colors distinguish adjacent chromosomes. The horizontal dashed line indicates the genome-wide significance threshold of *p* < 5 × 10^−8^. Multiple genome-wide significant loci were identified, suggesting that the latent airway disease factor captures common genetic susceptibility across clinically distinct airway disorders. The three panels represent complementary levels of genetic analysis: panel A summarizes pairwise genetic overlap, panel B evaluates whether the complete covariance structure can be represented by a common latent factor and quantifies the contribution of each disorder, and panel C identifies SNP associations with the shared gAirwayDisease liability.

We next tested whether the genetic covariance matrix of the four airway diseases supported a common latent factor. The overall KMO value was 0.808, and parallel analysis supported extraction of a single component. Exploratory factor analysis showed positive loadings for all four diseases, with the highest loading for bronchiectasis and the lowest for CRSsNP; full KMO, uniqueness, and exploratory factor analysis results are provided in Table S3. We then fitted a one-factor Genomic SEM model to define a shared airway disease latent factor, termed gAirwayDisease. All four diseases loaded positively onto the common factor (Figure 3B), with standardized factor loadings of 0.81 for COPD, 0.84 for asthma, 0.83 for bronchiectasis, and 0.66 for CRSsNP. Although CRSsNP had the lowest standardized loading (0.66), this remained a substantial contribution, corresponding to approximately 44% of its standardized genetic variance being captured by gAirwayDisease. Its lower loading was consistent with the comparatively weaker genetic correlations between CRSsNP and the three lower-airway diseases, suggesting that CRSsNP shares common airway disease liability while retaining a larger trait-specific genetic component. The model showed excellent fit (χ^2^ = 1.152, df = 2, *p* = 0.562, CFI = 1.000, SRMR = 0.032), supporting a shared genetic liability structure across these clinically distinct airway diseases. Complete confirmatory factor analysis estimates are provided in Table S4. Pairwise LDSC quantified six separate bivariate genetic relationships, whereas the Genomic SEM model evaluated whether the complete genetic covariance structure across the four disorders could be represented by a single latent factor. The standardized factor loadings quantified the relative contribution of each disease to this shared liability, while the residual genetic variances represented disease-specific components not captured by gAirwayDisease. The subsequent multivariate GWAS estimated SNP effects on the shared gAirwayDisease liability rather than on any individual disorder, providing a common genetic phenotype for downstream functional genomic analyses. Genome-wide association analysis of gAirwayDisease identified multiple loci exceeding the genome-wide significance threshold of *p* < 5 × 10^−8^ (Figure 3C). The Manhattan plot showed association peaks across several chromosomes, indicating that the latent factor captured shared genetic susceptibility across major airway diseases.

### 2.4. Spatial Mapping and Gene Prioritization of gAirwayDisease-Associated Signals

**Figure 4:**
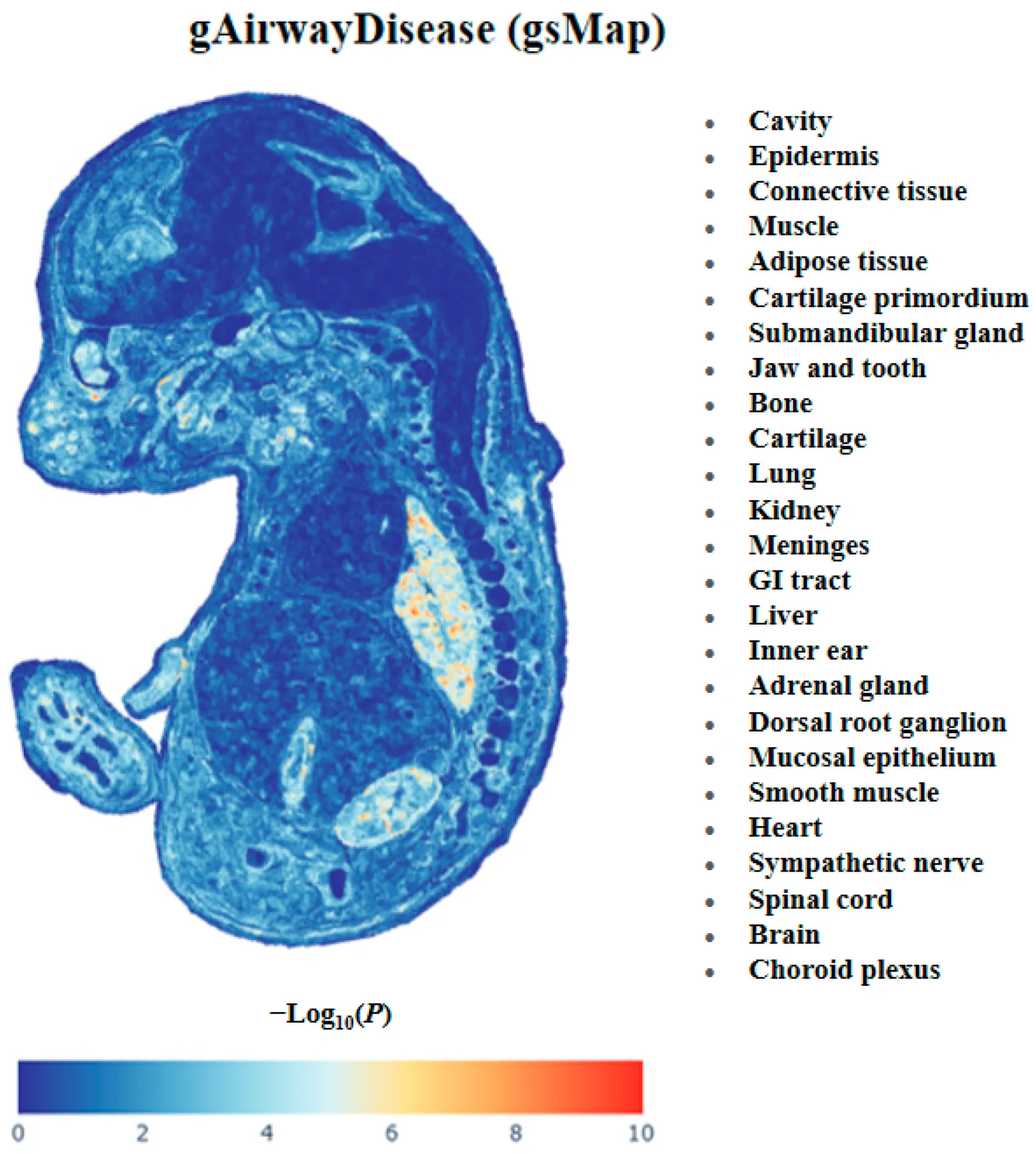
Anatomically resolved genetic enrichment of gAirwayDisease. gsMap analysis was performed to localize genetic signals for the Genomic SEM-derived gAirwayDisease factor across spatially resolved anatomical domains. gAirwayDisease integrates shared genetic liability across COPD, asthma, bronchiectasis and CRSsNP. The spatial map displays regional enrichment of trait-associated genetic signals on a sagittal anatomical reference section. Colors denote −log_10_(*p*) values from gsMap enrichment analysis, with blue indicating weak enrichment and yellow-to-red indicating stronger enrichment. Annotated tissue domains are listed on the right. Enrichment patterns highlight spatially localized airway- and epithelial-associated domains, supporting a coherent anatomical basis for the shared genetic architecture of chronic airway disease. The observed enrichment is consistent with the involvement of anatomical domains relevant to airway pathology but does not establish a direct functional mechanism.

**Figure 5:**
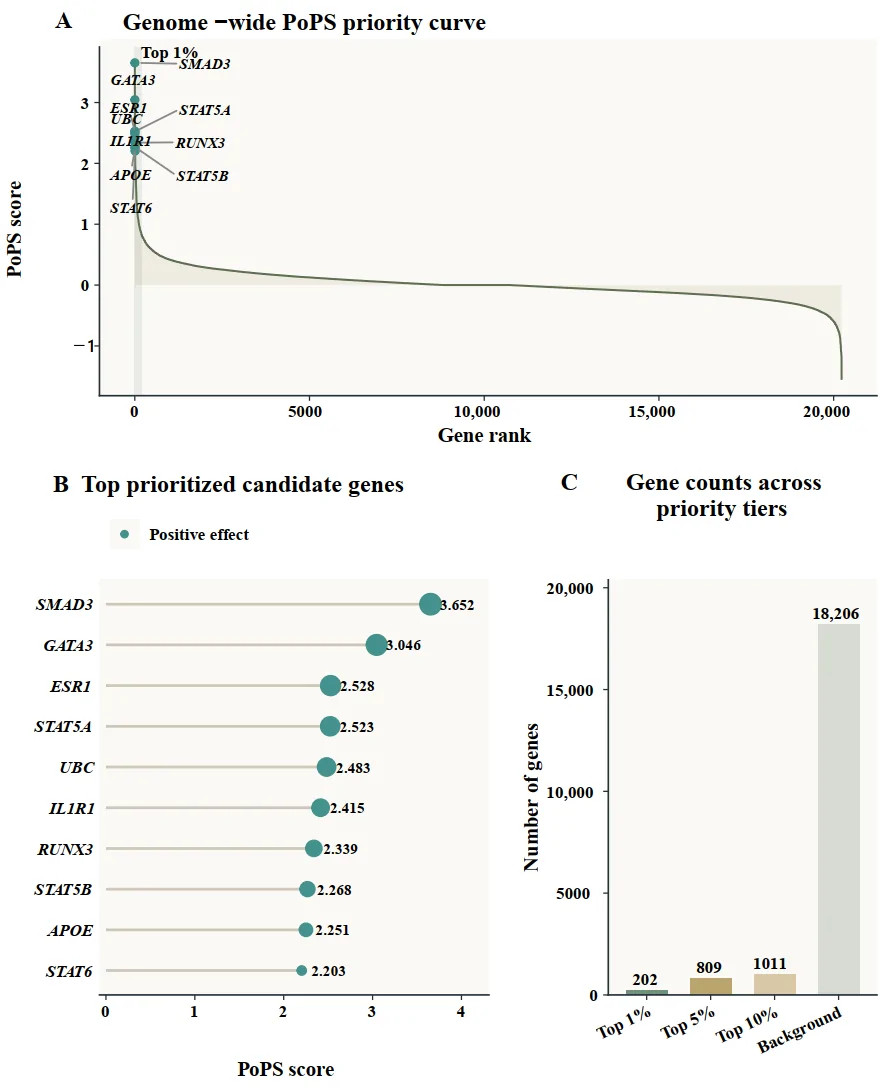
PoPS-based prioritization of candidate genes for shared airway disease liability. (**A**) Genome-wide PoPS priority curve for genes associated with the GSEM-derived gAirwayDisease factor. Genes are ranked according to their PoPS scores, with higher scores indicating stronger prioritization based on polygenic enrichment of gene-level features. The shaded region highlights the top 1% of prioritized genes, and the top ten genes are labeled. (**B**) Lollipop plot showing the top ten PoPS-prioritized candidate genes. Dot position represents the PoPS score, and dot size reflects the corresponding priority percentile. Colors indicate the direction of the gene-level effect where available. Gene symbols are shown in italic. (**C**) Summary of the number of genes assigned to each PoPS priority tier, including the top 1%, top 5%, top 10% and background gene sets. Together, these analyses prioritize candidate genes associated with the shared genetic architecture of major airway diseases for subsequent functional investigation.

We applied gsMap to localize gAirwayDisease-associated genetic signals across spatially resolved anatomical domains. The gsMap enrichment map showed heterogeneous spatial enrichment, with stronger signals reflected by higher −log_10_(*p*) values (Figure 4). Enrichment was concentrated in airway- and epithelial-associated anatomical domains, with additional signals extending to adjacent respiratory and connective tissue compartments. We next used PoPS to prioritize candidate effector genes for gAirwayDisease. The genome-wide priority curve showed a steep decline among the highest-ranked genes, indicating a restricted set of genes with markedly higher polygenic priority scores than the background distribution (Figure 5A). The top-ranked genes included *SMAD3*, *GATA3*, *ESR1*, *STAT5A*, *UBC*, *IL1R1*, *RUNX3*, *STAT5B*, *APOE*, and *STAT6*, with *SMAD3* showing the highest PoPS score, followed by *GATA3* and *ESR1* (Figure 5B). Together, these results prioritize immune-regulatory and airway-remodeling genes associated with shared airway disease liability.

### 2.5. MAGMA Gene-Set Enrichment Identifies Immune and Transcriptional Regulatory Pathways

MAGMA gene-set analysis highlights immune and transcriptional regulatory processes, with the strongest terms reaching approximately −log_10_ (adjusted *p*) = 6.7 (Figure S1). The enriched gene sets were dominated by immune activation and transcriptional regulatory processes, including B-cell activation, αβ T-cell activation, CD4-positive T-cell activation, regulation of T-cell proliferation, and *RUNX3*/*BCL11B*-related T-cell lineage transition. Additional enriched categories involved biosynthetic and RNA metabolic processes. Overall, the analysis showed enrichment of gene sets related to lymphocyte activation and transcriptional regulation in gAirwayDisease.

### 2.6. MTWAS Identifies Lung and Immune-Cell Expression Signals Associated with gAirwayDisease

We performed MTWAS to identify genetically regulated gene expression associated with gAirwayDisease. In GTEx v8 lung tissue analysis, the strongest positive signal was *SLC9A2* (Z = 6.955, *p* = 3.513 × 10^−12^, predicted R^2^ = 0.0679, Bonferroni *p* = 4.110 × 10^−9^, FDR *p* = 4.110 × 10^−9^), followed by *ORMDL3* (Z = 5.825, *p* = 5.704 × 10^−9^, predicted R^2^ = 0.0669, Bonferroni *p* = 6.286 × 10^−6^, FDR *p* = 2.095 × 10^−6^). Additional top positive lung signals included *SIPA1*, *RAPGEFL1*, *GNGT2*, *INTS12*, *CDK2AP1*, *EEFSEC*, *ZSWIM7*, and *HR*, with all top ten genes showing positive associations between genetically predicted lung expression and gAirwayDisease liability (Table 1). Immune-cell MTWAS using OneK1K weights identified recurrent cell-type-specific signals across CD4 T-cell, CD8 T-cell, and B-cell contexts (Table 2). The strongest and most recurrent signal was *IL18R1*, led by CD8 S100B cells (Z = 9.962, *p* = 2.226 × 10^−23^, Bonferroni *p* = 1.694 × 10^−20^), with additional strong associations in CD8 ET, CD4 NC, and CD8 NC cells. Other immune-cell signals included *PSMD3* in CD4 NC cells, *ORMDL3* in CD4 NC and CD8 NC cells, *HMG20A* in CD4 ET cells, and *CAMK4* across CD4 ET, CD4 NC, and B MEM cells. Complete GTEx v8 multi-tissue, UK Biobank-compatible GTEx v8, OneK1K, and DICE MTWAS results are provided in Tables S5–S8. These findings identify genetically predicted expression signals associated with gAirwayDisease but do not establish direct functional effects of the corresponding genes.

### 2.7. scMORE Identifies Immune-Cell-Specific Transcriptional Regulons Associated with gAirwayDisease

We next applied scMORE to identify transcription factor regulons associated with gAirwayDisease. Using prespecified criteria of CTS *p* < 0.05, GRS *p* < 0.05, and TRS *p* < 0.05, scMORE identified 36 significant regulon–cell type pairs (Table 3). These signals spanned pDCs, mDCs, B cells, CD14+ monocytes, FCGR3A+ monocytes, CD4+ T cells, CD8+ T cells, and NK cells. Prominent regulons included BCL11A, SPIB, and TCF4 in pDCs and B cells; KLF4 in mDCs and monocyte subsets; and ETS1, FOXO1, IKZF1, RUNX1, and STAT4 across T-cell and NK-cell populations. Literature-guided interpretation of the 36 significant regulon–cell type pairs indicated a mixture of non-Type 2-associated and shared immune regulatory programs. STAT4 regulons in CD4+ T cells, CD8+ T cells, and NK cells were consistent with Type 1-associated or cytotoxic immune regulation, whereas regulons identified in pDCs, B cells, and monocytes were mainly related to shared or lineage-specific immune regulation. GATA3- and STAT6-centered regulons were not among the significant scMORE findings under the prespecified criteria, although both genes were independently prioritized by PoPS. These findings identify coordinated immune-cell-specific regulatory programs associated with shared airway disease liability. Their potential relationships with epithelial dysfunction and airway remodeling are considered in the Discussion, but the results do not establish direct functional effects. The complete scMORE regulon results and literature-guided annotations are provided in Table S9.

### 2.8. MR-PheWAS Provides Phenotypic Context for gAirwayDisease

We performed phenome-wide Mendelian randomization using genetically predicted gAirwayDisease as the exposure. For the main MR-PheWAS visualization, we retained associations supported by more than five instrumental SNPs and Bonferroni-adjusted *p* < 0.05 (Figure S2; Table S10). Positive associations were observed for asthma (β = 2.270, 95% CI 2.067 to 2.474; 22 SNPs; Bonferroni-adjusted *p* = 2.93 × 10^−102^) and allergic rhinitis (β = 2.369, 95% CI 2.018 to 2.720; 41 SNPs; Bonferroni-adjusted *p* = 2.51 × 10^−36^). Higher genetically predicted gAirwayDisease liability was also associated with higher eosinophil counts (β = 1.206, 95% CI 0.871 to 1.541; 131 SNPs; Bonferroni-adjusted *p* = 7.20 × 10^−9^). Conversely, inverse associations were observed for FEV1/FVC (β = −1.540, 95% CI −1.852 to −1.228; 143 SNPs; Bonferroni-adjusted *p* = 1.74 × 10^−18^) and FEV1 (β = −0.916, 95% CI −1.179 to −0.652; 98 SNPs; Bonferroni-adjusted *p* = 4.13 × 10^−8^). These findings provide complementary phenotypic context showing that gAirwayDisease is associated with respiratory, allergic, lung function, and immune-related traits.

## 3. Discussion

This study used Genomic SEM to define gAirwayDisease, a latent genetic factor capturing shared liability across COPD, asthma, bronchiectasis and CRSsNP. By integrating spatial genetic mapping, PoPS-based gene prioritization, MAGMA gene-set enrichment, lung and immune-cell MTWAS, scMORE regulon analysis, and phenome-wide Mendelian randomization, we characterized multiple functional genomic features associated with shared airway disease susceptibility. Across these analytical layers, airway epithelial domains, lung-expressed genes, and immune-cell-specific regulatory programs were consistently prioritized. Because these genes were prioritized from analyses of gAirwayDisease rather than from four separate single-trait associations, they should be interpreted as candidate contributors to the genetic covariance shared across the four disorders, rather than as genes proven to exert identical effects in each disease.

The positive genetic correlations among COPD, asthma, bronchiectasis, and CRSsNP support a shared polygenic basis across major airway diseases [6,16,17]. Although these disorders differ in anatomical distribution, age of onset, and clinical presentation, they share key pathological features, including chronic inflammation, epithelial dysfunction, mucus dysregulation, and airway remodeling [18,19]. Pairwise genetic correlations provide evidence of overlap between disease pairs, but they do not define a unified liability factor across multiple airway diseases. Genomic SEM addresses this limitation by modeling the full genetic covariance matrix and deriving latent factors that capture shared genetic architecture across complex traits [7]. In our analysis, all four diseases loaded positively onto gAirwayDisease, with standardized loadings ranging from 0.66 for CRSsNP to 0.84 for asthma. The lower loading of CRSsNP may reflect both its upper-airway anatomical context and its substantial inflammatory heterogeneity. CRSsNP can encompass Type 1, Type 2, Type 3, and mixed inflammatory endotypes, whereas the shared factor was more strongly defined by the three lower-airway disorders. Thus, the loading of 0.66 does not indicate an absence of shared genetic liability, but suggests that CRSsNP also retains a larger upper-airway-specific genetic component. The strong loadings of COPD, asthma, and bronchiectasis indicate that lower-airway diseases contributed substantially to the shared factor, whereas the positive loading of CRSsNP extends this shared liability to upper-airway inflammatory disease. These findings support a genetically informed model of airway disease continuity across anatomical compartments and extend previous applications of Genomic SEM to respiratory medicine [7,8]. Together, they suggest that shared airway disease liability can be modeled as a biologically interpretable genetic construct rather than as a collection of pairwise overlaps.

Pairwise LDSC and Genomic SEM provided complementary rather than redundant information. Pairwise genetic correlations established that each disease pair shared a proportion of its polygenic architecture but could not determine whether the six pairwise relationships reflected a coherent common genetic dimension. By jointly modeling the complete genetic covariance matrix, Genomic SEM showed that the four disorders could be represented by a well-fitting latent factor and quantified the relative contribution and residual genetic component of each disease. Moreover, the multivariate GWAS of gAirwayDisease identified genetic associations with the shared liability, allowing the downstream gsMap, PoPS, MTWAS, and scMORE analyses to focus on genetic features common across the airway disease spectrum rather than those associated with a single disorder. Thus, gAirwayDisease is not simply an average of the four phenotypes, but a model-derived genetic construct representing their covariance. Nevertheless, the different factor loadings and residual variances indicate that substantial disease-specific genetic heterogeneity remains.

Spatial genetic mapping localized gAirwayDisease-associated signals to airway- and epithelial-associated anatomical domains. This finding is consistent with the central role of the airway epithelium as both a physical barrier and an immune-active interface in chronic respiratory disease [18,19]. The airway epithelium senses environmental exposures, supports mucociliary clearance, secretes cytokines and chemokines, and coordinates immune-cell recruitment. Epithelial dysfunction therefore provides a plausible anatomical and cellular substrate through which shared genetic risk may contribute to multiple airway diseases. Epithelial barrier impairment has been reported in both asthma and COPD. Airway epithelial cultures from patients with asthma show reduced barrier integrity and increased permeability compared with controls [20]. Type 2 cytokines, including IL-4 and IL-13, can disrupt epithelial tight junction integrity, and ILC2-derived IL-13 may further impair epithelial barrier function [21,22]. In COPD, chronic oxidative stress and inflammation contribute to epithelial injury, cellular senescence, and abnormal repair [18]. In this context, the gsMap results provide anatomical coherence to the Genomic SEM-derived factor, indicating that gAirwayDisease maps to tissue domains directly relevant to airway pathology.

Lung MTWAS prioritized *SLC9A2* and *ORMDL3* as the strongest lung-expressed genes whose genetically predicted expression was positively associated with gAirwayDisease. Because these associations were derived from the gAirwayDisease factor rather than from an individual disease GWAS, they should be interpreted as candidate contributors to the genetic covariance shared across the four airway disorders, rather than as genes proven to exert identical effects in each disease. *ORMDL3* is notable because the 17q21 locus regulating its expression is one of the most reproducible asthma susceptibility regions [23,24]. Functional studies have linked *ORMDL3* to sphingolipid metabolism, ceramide homeostasis, cellular inflammation, mucus production, and allergic airway inflammation [23,24,25,26]. Its identification in both lung and immune-cell MTWAS provides cross-tissue genetic support for its relevance to shared airway disease susceptibility. An *ORMDL3* polymorphism has also been associated with both asthma and COPD, suggesting that its genetic relevance may extend beyond a single airway disorder. Immune-cell MTWAS identified *IL18R1* as the strongest and most recurrent signal across CD4 and CD8 T-cell subsets. *IL18R1* encodes interleukin-18 receptor 1, and IL-18 is a proinflammatory cytokine involved in immune activation and inflammatory disease pathogenesis [27]. Increased IL-18 and IL-18Rα expression has also been reported in allergic asthma-related immune contexts [28]. Large-scale asthma GWAS have implicated the *IL1RL1*/*IL18R1* locus, while cross-trait analyses have identified *IL18R1* and *IL1R1* as pleiotropic genes shared between COPD and allergic diseases [6]. Together with the prioritization of *IL1R1* by PoPS, the recurrent *IL18R1* associations across T-cell subsets support IL-1/IL-18-related cytokine signaling as a plausible immune component of common airway disease liability. Nevertheless, these convergent genetic findings require functional validation and do not establish direct mechanisms or identical effects across all four disorders.

PoPS also prioritized *GATA3*, *STAT6*, *RUNX3*, and *SMAD3*, indicating complementary immune and structural components of shared airway disease liability. *GATA3* and *STAT6* regulate Type 2 lymphocyte differentiation and IL-4/IL-13 signaling, whereas *RUNX3* contributes to T-cell lineage regulation and the balance between Type 1 and Type 2 immune responses [29,30,31]. Their joint prioritization suggests that gAirwayDisease captures broader immune dysregulation rather than an exclusively Type 2 inflammatory program. *SMAD3* further links the shared genetic factor to transforming growth factor-β signaling and airway remodeling, including fibroblast activation, extracellular matrix deposition, and structural airway changes [32,33,34,35]. Collectively, these genes connect gAirwayDisease to epithelial dysfunction, immune activation, and airway remodeling, which are shared pathological features across the four airway disorders. Nevertheless, these results prioritize biologically plausible candidates and do not demonstrate that each gene exerts identical effects across all four diseases.

The scMORE analysis extended the gene-level findings by identifying coordinated transcriptional programs across several immune-cell populations. *BCL11A*, *SPIB*, and *TCF4* regulons in plasmacytoid dendritic cells and B cells point to regulatory programs involved in dendritic-cell identity, antigen sensing, and humoral immune activation [36,37,38]. *KLF4* regulons in dendritic cells and monocytes suggest the involvement of myeloid-cell state regulation and innate inflammatory responses, whereas *ETS1*, *FOXO1*, *IKZF1*, *RUNX1*, and *STAT4* regulons across T-cell and NK-cell populations highlight lymphocyte differentiation, cytokine responsiveness, and Type 1-associated or cytotoxic immune activity [39]. These findings indicate that the shared genetic liability involves coordinated innate and adaptive immune regulation rather than a single immune pathway. Although scMORE did not directly assess airway epithelial cells, these immune regulatory programs may be relevant to epithelial dysfunction and airway remodeling through immune–epithelial crosstalk. Cytokines and inflammatory mediators released by dendritic cells, monocytes, T cells, and NK cells can influence epithelial barrier integrity, mucus production, tissue injury, and repair [18,19,20,21,22]. The scMORE findings therefore complement the epithelial enrichment observed in gsMap and the lung-expression signals identified by MTWAS. Together, these findings highlight dendritic-cell, B-cell, T-cell, and NK-cell regulatory programs associated with shared airway disease liability. They suggest coordinated regulatory signals across multiple immune-cell populations but do not demonstrate that these regulons directly mediate disease development. The regulatory analyses provided different levels of anatomical resolution. Lung MTWAS primarily captured lower-airway tissue expression, whereas OneK1K and scMORE identified immune-cell programs that were not specific to either airway compartment. The present findings therefore support shared epithelial and immune involvement but do not directly establish regulatory differences between upper- and lower-airway epithelial cells. Such comparisons will require matched upper- and lower-airway single-cell and genetic regulatory datasets.

The MR-PheWAS analysis provided phenome-wide clinical context for genetically predicted gAirwayDisease. Significant associations clustered around respiratory disease, allergic disease, impaired lung function, and immune-cell traits. Positive associations with asthma- and allergic rhinitis-related outcomes were consistent with the inclusion of asthma and CRSsNP in the shared airway disease factor, whereas negative associations with FEV1- and FVC-related traits indicated alignment with clinically relevant respiratory impairment. These findings should not be interpreted as definitive causal evidence for individual outcomes because of potential horizontal pleiotropy, weak instruments, and phenotype heterogeneity. Instead, they provide complementary phenotypic context indicating that gAirwayDisease captures respiratory, allergic, lung function, and immune-related liability.

This study has several strengths. First, by applying Genomic SEM, we moved beyond pairwise genetic correlations to define a latent genetic liability factor shared across four major airway diseases. Second, we integrated complementary genetic, spatial, transcriptomic, immune-cell and regulon-level analyses, enabling biological interpretation of gAirwayDisease across anatomical, epithelial and immune regulatory contexts. Third, independent analytical layers converged on airway epithelial domains, immune activation pathways, lung-expressed genes and immune-cell regulatory programs, strengthening the biological plausibility of the shared liability model. Several limitations should be acknowledged. First, all GWAS summary statistics used to construct gAirwayDisease were derived from European-ancestry cohorts. Differences in allele frequencies and linkage disequilibrium patterns across populations may limit the transferability of the estimated factor structure, associated loci, and prioritized genes. The present findings should therefore be interpreted within a European-ancestry context and require validation in ancestrally diverse populations. Second, the analysis was based on GWAS summary statistics, precluding direct adjustment for clinical covariates, smoking, medication use, environmental exposures, disease severity and longitudinal trajectories. Third, the input GWAS differed in phenotype definition, case ascertainment, cohort composition and sample size, with bronchiectasis having a smaller effective sample size than the other traits. Fourth, the functional interpretations derived from gsMap, PoPS, MAGMA, MTWAS, and scMORE are based on statistical genetic and functional genomic integration rather than direct experimental testing. These analyses prioritize candidate tissues, genes, enriched gene sets, and regulatory programs but do not establish their cellular functions or disease mechanisms. Experimental perturbation studies in airway epithelial and relevant immune-cell models, followed by validation in disease-relevant airway tissues or animal models, will be required to determine their biological roles. Finally, formal gene–environment interactions between gAirwayDisease and smoking, allergens, or other environmental exposures could not be evaluated because individual-level exposure data and exposure-stratified GWAS interaction estimates were unavailable. The MR-PheWAS and genetic association results should not be interpreted as tests of interaction. Future studies integrating individual-level genetic data with harmonized smoking and allergen exposure measurements are needed to determine whether environmental exposures modify the genetic effects captured by gAirwayDisease.

## 4. Materials and Methods

### 4.1. GWAS Summary Statistics for Airway Diseases

We included GWAS summary statistics for four major airway diseases, all derived from participants of European genetic ancestry: COPD, asthma, bronchiectasis, and CRSsNP. The COPD GWAS was obtained from the GWAS Catalog under accession GCST90668057 and included 16,712 cases and 389,099 controls [40]. Asthma GWAS summary statistics were obtained from the Global Biobank Meta-analysis Initiative under GWAS Catalog accession GCST90399686 and included 121,940 cases and 1,254,131 controls of European ancestry [41]. European-specific bronchiectasis GWAS summary statistics were downloaded from PheWeb.jp and were derived from the study reported by Sakaue, Kanai, and colleagues [42]. CRSsNP GWAS summary statistics were obtained from GWAS Catalog accession GCST90652532 and included 15,448 cases and 685,602 controls of European ancestry [5].

For binary traits with available case–control information, effective sample size was calculated as Neff = 4 × Ncase × Ncontrol/(Ncase + Ncontrol). Summary statistics were harmonized to a common format containing chromosome, base-pair position, SNP identifier, effect allele, non-effect allele, effect allele frequency, effect estimate, standard error, association *p* value, and sample size. Effect directions were aligned so that positive effects corresponded to increased liability to each airway disease. Additional details on GWAS harmonization, genetic correlation analysis, Genomic SEM modeling, functional annotation, MTWAS, scMORE regulon analysis, and phenome-wide Mendelian randomization are provided in the eMethods.

### 4.2. Global and Local Genetic Correlation Analyses

We first evaluated the SNP-based heritability and global genetic correlation structure of the four airway disease GWAS datasets using linkage disequilibrium score regression (LDSC) [43]. Univariate LDSC was used to estimate observed-scale SNP heritability, genomic inflation metrics and LDSC intercepts for each trait. Bivariate LDSC was then used to estimate pairwise genetic covariances and genetic correlations among COPD, asthma, bronchiectasis and CRSsNP. Bonferroni correction was applied across the six pairwise genetic correlation tests. To further characterize regional heterogeneity in shared genetic architecture, we performed local genetic correlation analysis using Local Analysis of [co]Variant Association (LAVA, version 0.1.5) [44]. Pairwise local genetic correlations were estimated across semi-independent genomic regions for each airway disease pair. Local correlation signals were visualized using locus-level volcano plots, and multiple testing was controlled using the Benjamini–Hochberg false discovery rate procedure.

### 4.3. Genomic SEM and Construction of gAirwayDisease

We applied Genomic SEM to model the shared genetic covariance structure across COPD, asthma, bronchiectasis and CRSsNP [7]. Multivariable LDSC was used to estimate the genetic covariance and sampling covariance matrices across the four traits. The suitability of the genetic covariance matrix for latent-factor modeling was assessed using the Kaiser–Meyer–Olkin criterion, parallel analysis and exploratory factor analysis. A one-factor common-factor model was then fitted, defining a latent shared airway disease factor, termed gAirwayDisease.

Model fit was assessed using χ^2^, comparative fit index and standardized root mean square residual. Standardized factor loadings were used to evaluate the contribution of each airway disease to the shared latent factor. A genome-wide association analysis of the latent gAirwayDisease factor was subsequently performed within the Genomic SEM framework. Genome-wide significance was defined as *p* < 5 × 10^−8^. Variants in the extended major histocompatibility complex region were excluded from downstream visualization and post-GWAS interpretation where appropriate because of its complex linkage disequilibrium structure.

### 4.4. Functional Annotation, Spatial Mapping and Gene Prioritization

We performed spatial and gene-level analyses to interpret the biological context of gAirwayDisease-associated genetic signals. First, gsMap was used to integrate gAirwayDisease GWAS summary statistics with spatial transcriptomic reference data, enabling anatomically resolved enrichment analysis of trait-associated genetic signals [45]. Second, the polygenic priority score (PoPS) framework was used to prioritize candidate genes by leveraging genome-wide polygenic signal and gene-level functional features [10]. Third, MAGMA was used for gene-set enrichment analysis to identify biological pathways and immune regulatory processes enriched among gAirwayDisease-associated genes [46]. Together, these analyses were used to characterize anatomical enrichment, prioritize candidate genes, and identify enriched gene sets associated with shared airway disease liability.

### 4.5. Transcriptome-Wide Association Analyses

We performed MTWAS analyses to identify genetically regulated gene expression associated with gAirwayDisease [47]. Four complementary MTWAS settings were evaluated. First, we performed GTEx v8-based multi-tissue MTWAS across human tissues to assess the broader tissue-expression architecture of gAirwayDisease. Second, we focused on lung tissue using UK Biobank-compatible GTEx v8 MTWAS prediction weights, in which eQTL SNPs were restricted to variants available in UK Biobank GWAS datasets to improve compatibility with UK Biobank-derived phenotypes [47,48]. Positive MTWAS Z values indicated that higher genetically predicted expression was associated with increased gAirwayDisease liability. To further evaluate immune-cell-specific genetically regulated expression, we performed MTWAS using OneK1K immune-cell expression prediction weights [14,47]. As a sensitivity analysis, we repeated immune-cell MTWAS using DICE-based weights, which capture immune-cell and activation-state-specific regulatory effects [15,47].

### 4.6. scMORE Regulon Analysis

To investigate cell-type-specific transcriptional regulatory programs associated with shared airway disease liability, we applied single-cell multiomics regulon enrichment (scMORE) to the gAirwayDisease GWAS summary statistics [49]. scMORE integrates GWAS summary statistics with single-cell multiomic regulatory information to identify cell-type-specific transcription factor regulons, also referred to as eRegulons, that are supported by disease-associated genetic signals. Regulons were evaluated using cell-type specificity, genomic risk support and total regulon score. Positive regulon results were defined by simultaneous evidence of CTS *p* < 0.05, GRS *p* < 0.05 and TRS *p* < 0.05.

### 4.7. Phenome-Wide Mendelian Randomization

Finally, we performed phenome-wide two-sample Mendelian randomization to examine whether genetically predicted gAirwayDisease was associated with downstream clinical, immune and molecular traits. Outcome GWAS datasets were obtained from MR-Base and related TwoSampleMR-compatible resources after excluding protein, gene, metabolite and imaging traits where appropriate [50]. Harmonization and MR analyses were conducted using TwoSampleMR-compatible workflows. Wald ratio estimates were used for single-SNP instruments, whereas inverse-variance weighted estimates were used when multiple SNP instruments were available. Multiple testing was controlled using Bonferroni correction. For visualization, we focused on associations supported by more than five instrumental SNPs and Bonferroni-adjusted *p* < 0.05.

## 5. Conclusions

This study defines gAirwayDisease as a latent genetic factor capturing shared liability across COPD, asthma, bronchiectasis, and CRSsNP within European-ancestry GWAS datasets. Supported by genome-wide genetic correlations and a well-fitting Genomic SEM model, gAirwayDisease converged on airway epithelial domains, lung-expressed genes, immune-cell expression signals, and transcriptional regulons. Phenome-wide MR further linked gAirwayDisease to respiratory, allergic, lung function, and immune-related traits. Together, these findings provide a hypothesis-generating functional genomic framework for shared airway disease liability and prioritize epithelial and immune regulatory candidates for future experimental validation.

## Figures and Tables

**Table 1:** GTEx v8 lung MTWAS results for gAirwayDisease.

Gene	MTWAS *Z*	MWAS *p*	Predicted R^2^	Prediction *p*	TWAS Bonferroni *p*	TWAS FDR *p*	Tissue
*SLC9A2*	6.955	3.513 × 10^−12^	0.0679	2.085 × 10^−5^	4.110 × 10^−9^	4.110 × 10^−9^	Lung
*ORMDL3*	5.825	5.704 × 10^−9^	0.0669	6.118 × 10^−5^	6.286 × 10^−6^	2.095 × 10^−6^	Lung
*SIPA1*	5.381	7.406 × 10^−8^	0.0291	1.797 × 10^−3^	9.109 × 10^−5^	9.109 × 10^−5^	Lung
*RAPGEFL1*	5.287	1.245 × 10^−7^	0.215	<2.2 × 10^−16^	1.373 × 10^−4^	2.745 × 10^−5^	Lung
*GNGT2*	5.132	2.864 × 10^−7^	0.0724	2.178 × 10^−8^	3.157 × 10^−4^	3.946 × 10^−5^	Lung
*INTS12*	5.064	4.103 × 10^−7^	0.0927	4.112 × 10^−9^	2.868 × 10^−4^	2.868 × 10^−4^	Lung
*CDK2AP1*	4.819	1.443 × 10^−6^	0.0808	1.085 × 10^−8^	1.400 × 10^−3^	3.500 × 10^−4^	Lung
*EEFSEC*	4.805	1.549 × 10^−6^	0.0537	2.061 × 10^−3^	1.554 × 10^−3^	1.554 × 10^−3^	Lung
*ZSWIM7*	4.55	5.374 × 10^−6^	0.0996	2.561 × 10^−10^	5.922 × 10^−3^	5.384 × 10^−4^	Lung
*HR*	4.484	7.322 × 10^−6^	0.0297	1.021 × 10^−3^	4.627 × 10^−3^	1.157 × 10^−3^	Lung

Footnote. MTWAS used GTEx v8 lung expression prediction weights. Positive Z indicates higher genetically predicted lung expression associated with increased gAirwayDisease liability. MWAS *p* is the nominal association *p* value; Bonferroni and FDR *p* values are multiple-testing-adjusted.

**Table 2:** OneK1K immune-cell MTWAS results for gAirwayDisease.

Gene	MTWAS *Z*	MWAS *p*	Predicted R^2^	Prediction *p*	TWAS Bonferroni *p*	TWAS FDR *p*	Tissue
*IL18R1*	9.962	2.226 × 10^−23^	0.0125	0.0163	1.694 × 10^−20^	1.694 × 10^−20^	CD8 S100B
*IL18R1*	9.826	8.724 × 10^−23^	0.037	6.477 × 10^−6^	8.148 × 10^−20^	8.148 × 10^−20^	CD8 ET
*IL18R1*	9.033	1.675 × 10^−19^	0.0721	2.109 × 10^−15^	1.621 × 10^−16^	1.621 × 10^−16^	CD4 NC
*IL18R1*	8.722	2.737 × 10^−18^	0.0127	0.00566	2.516 × 10^−15^	2.516 × 10^−15^	CD8 NC
*PSMD3*	7.376	1.629 × 10^−13^	0.0215	8.340 × 10^−5^	1.546 × 10^−10^	5.350 × 10^−11^	CD4 NC
*ORMDL3*	5.825	5.704 × 10^−9^	0.267	<2.2 × 10^−16^	5.413 × 10^−6^	1.083 × 10^−6^	CD4 NC
*ORMDL3*	5.825	5.704 × 10^−9^	0.1528	<2.2 × 10^−16^	5.122 × 10^−6^	2.561 × 10^−6^	CD8 NC
*HMG20A*	5.735	9.767 × 10^−9^	0.011	0.00275	3.555 × 10^−6^	3.555 × 10^−6^	CD4 ET
*CAMK4*	5.497	3.869 × 10^−8^	0.0246	0.000235	2.097 × 10^−5^	6.990 × 10^−6^	CD4 ET
*CAMK4*	5.497	3.869 × 10^−8^	0.0107	0.0196	2.426 × 10^−5^	1.213 × 10^−5^	CD4 NC
*CAMK4*	5.497	3.869 × 10^−8^	0.0117	0.00509	2.039 × 10^−5^	2.039 × 10^−5^	B MEM

Footnote. Top immune-cell MTWAS signals using OneK1K prediction weights. Positive Z indicates higher genetically predicted immune-cell expression associated with increased gAirwayDisease liability.

**Table 3:** scMORE regulons associated with gAirwayDisease.

Regulon	Cell Type	CTS	CTS *p*	GRS	GRS *p*	TRS	TRS *p*
*BCL11A*	pDCs	2.383	0.01	4.232	<0.001	2.628	0.006
*SPIB*	pDCs	3.521	<0.001	3.781	<0.001	3.757	<0.001
*TCF4*	pDCs	3.028	<0.001	3.34	<0.001	3.251	<0.001
*ZNF521*	pDCs	2.908	0.006	3.112	0.003	3.267	<0.001
*KLF4*	mDCs	1.825	0.011	4.513	<0.001	2.083	0.007
*BCL11A*	B cells	3.805	<0.001	4.168	<0.001	3.922	<0.001
*EBF1*	B cells	3.293	<0.001	3.34	<0.001	3.018	<0.001
*IKZF3*	B cells	1.754	0.024	1.913	0.02	1.856	0.019
*JAZF1*	B cells	1.753	0.025	3.429	<0.001	1.919	0.012
*POU2F2*	B cells	2.257	0.003	1.642	0.048	2.35	0.002
*SPIB*	B cells	3.229	<0.001	3.648	<0.001	3.39	<0.001
*TCF4*	B cells	2.319	0.002	3.359	<0.001	2.561	<0.001
*TRERF1*	B cells	1.787	0.018	1.946	0.023	1.959	0.004
*IKZF1*	CD14+ monocytes	1.888	0.003	2.627	0.005	1.622	<0.001
*KLF4*	CD14+ monocytes	2.04	<0.001	4.322	<0.001	2.026	<0.001
*IKZF1*	FCGR3A+ monocytes	1.782	0.013	2.588	0.007	1.9	0.007
*KLF4*	FCGR3A+ monocytes	1.874	0.011	4.463	<0.001	2.059	0.002
*ETS1*	CD4+ T cells	2.378	0.006	2.676	0.002	2.49	0.003
*FOXO1*	CD4+ T cells	3.379	<0.001	2.212	0.01	3.473	<0.001
*HIVEP2*	CD4+ T cells	3.139	<0.001	1.659	0.044	3.204	<0.001
*IKZF1*	CD4+ T cells	2.918	<0.001	2.627	0.003	3.012	<0.001
*RUNX1*	CD4+ T cells	3.534	<0.001	3.502	<0.001	3.688	<0.001
*STAT4*	CD4+ T cells	2.477	<0.001	1.879	0.027	2.555	<0.001
*ETS1*	CD8+ T cells	2.978	<0.001	2.765	0.004	3.111	<0.001
*FOXO1*	CD8+ T cells	3.658	<0.001	2.239	0.008	3.766	<0.001
*HIVEP2*	CD8+ T cells	3.187	<0.001	1.781	0.04	3.265	<0.001
*IKZF1*	CD8+ T cells	3.204	<0.001	2.67	0.005	3.322	<0.001
*NFATC3*	CD8+ T cells	2.017	0.032	1.696	0.046	2.109	0.026
*RUNX1*	CD8+ T cells	3.8	<0.001	3.568	<0.001	3.979	<0.001
*STAT4*	CD8+ T cells	2.838	<0.001	1.925	0.028	2.933	<0.001
*ETS1*	NK cells	1.829	0.039	2.753	0.002	1.951	0.028
*FOXO1*	NK cells	1.71	0.05	2.154	0.018	1.805	0.037
*GTF3A*	NK cells	1.908	0.031	1.644	0.047	2.005	0.024
*HIVEP2*	NK cells	1.81	0.041	1.712	0.046	1.881	0.034
*RUNX1*	NK cells	2.321	0.011	3.492	<0.001	2.485	0.006
*STAT4*	NK cells	2.776	<0.001	1.912	0.036	2.858	<0.001

Footnote. Significant regulon–cell type pairs were defined by CTS *p* < 0.05, GRS *p* < 0.05, and TRS *p* < 0.05.

## Data Availability

All data used in this study were obtained from publicly available GWAS summary statistics and functional genomics resources. COPD GWAS summary statistics were obtained from the GWAS Catalog under accession GCST90668057. Asthma GWAS summary statistics were obtained from the Global Biobank Meta-analysis Initiative through GWAS Catalog accession GCST90399686. Bronchiectasis GWAS summary statistics were downloaded from PheWeb.jp as GWASsummary_Bronchiectasis_EUR_SakaueKanai2020.auto.txt.gz. CRSsNP GWAS summary statistics were obtained from the GWAS Catalog under accession GCST90652532. GTEx v8, OneK1K, and DICE expression prediction weights were obtained from the MTWAS resource. Phenome-wide Mendelian randomization outcome datasets were obtained from MR-Base and related TwoSampleMR-compatible resources. Processed result tables are provided in the Supplementary Materials. Derived gAirwayDisease GWAS summary statistics and analysis scripts are available from the corresponding author upon reasonable request.

## Supplementary Materials

The following supporting information can be downloaded at https://www.mdpi.com/article/10.3390/ijms27167450/s1.
