# Supplementary material for: Genetic Evidence for Unified Airway Disease: Shared Epithelial and Immune Architecture Across Major Airway Diseases
Source: Int J Mol Sci. 2026 Aug 20;27(16):7450. doi: 10.3390/ijms27167450 (PMC13513354; doi:10.3390/ijms27167450)
Supplement: Supplementary file 1 [file ijms-27-07450-s001.zip › ijms-4455150-supplementary.pdf]

Genetic evidence for unified airway disease: Shared immune architecture across asthma and inflammatory disorders

Contents

Supplementary Methods....2
Supplementary Methods 1. GWAS dataset selection and source description 2
Supplementary Methods 2. GWAS summary statistic harmonization 2
Supplementary Methods 3. LDSC-based SNP heritability and global genetic correlation 3
Supplementary Methods 4. Local genetic correlation analysis using LAVA 3
Supplementary Methods 5. Assessment of factorability before Genomic SEM 4
Supplementary Methods 6. Genomic SEM and construction of gAirwayDisease....4
Supplementary Methods 7. Manhattan plot visualization of gAirwayDisease GWAS 5
Supplementary Methods 8. gsMap spatial enrichment analysis....5
Supplementary Methods 9. PoPS gene prioritization....5
Supplementary Methods 10. MAGMA gene-set enrichment analysis 6
Supplementary Methods 11. GTEx v8 lung MTWAS analysis....6
Supplementary Methods 12. OneK1K immune-cell MTWAS analysis....7
Supplementary Methods 13. DICE immune-cell MTWAS sensitivity analysis....7
Supplementary Methods 14. scMORE regulon enrichment analysis....8
Supplementary Methods 15. Phenome-wide Mendelian randomization....8
Supplementary Methods 16. Software and statistical reporting....9
References....9
Supplementary Results....11
Supplementary Results 1. GWAS architecture and LDSC quality metrics 11
Supplementary Results 2. Pairwise genetic covariance and genetic correlation....11
Supplementary Results 3. Local genetic correlation patterns 11
Supplementary Results 4. Factorability assessment and exploratory factor analysis....12
Supplementary Results 5. Confirmatory Genomic SEM model 12
Supplementary Results 6. Spatial mapping and PoPS prioritization....12
Supplementary Results 7. MAGMA gene-set enrichment 13
Supplementary Results 8. GTEx v8 MTWAS across tissues 13
Supplementary Results 9. OneK1K and DICE immune-cell MTWAS....13
Supplementary Results 10. scMORE regulon enrichment....14
Supplementary Results 11. Phenome-wide Mendelian randomization 14

Supplementary Methods

Supplementary Methods 1. GWAS dataset selection and source description

GWAS summary statistics were obtained for four major airway diseases: chronic obstructive pulmonary disease (COPD), asthma, bronchiectasis and chronic rhinosinusitis without nasal polyps (CRSsNP). These diseases were selected because they represent clinically important airway disorders with partially overlapping inflammatory, epithelial and mucus-related disease mechanisms, while also covering both lower and upper airway phenotypes.

COPD summary statistics were obtained from GWAS Catalog accession GCST90668057. This GWAS was derived from a multi-domain rule-based phenotyping algorithm for COPD in UK Biobank and included 16,712 cases and 389,099 controls. The original GWAS was performed using genome-wide genotyping array data and adjusted for age, sex, genotyping array and the top 20 genetic principal components [1]. In the present study, the COPD GWAS was used to represent genetic liability to chronic obstructive pulmonary disease.

Asthma summary statistics were obtained from GWAS Catalog accession GCST90399686, corresponding to the Global Biobank Meta-analysis Initiative asthma GWAS. This dataset included 121,940 European-ancestry asthma cases and 1,254,131 controls and was generated from a large-scale biobank meta-analysis across human diseases [2]. The asthma GWAS was used to represent genetic liability to asthma.

Bronchiectasis summary statistics were downloaded from PheWeb.jp as GWASsummary_Bronchiectasis_EUR_SakaueKanai2020.auto.txt.gz. This file corresponds to the European-ancestry bronchiectasis summary statistics from the cross-population atlas of genetic associations for 220 human phenotypes reported by Sakaue, Kanai and colleagues [3]. Because case-control counts and maximum sample size were not available in the analysis notes used here, the bronchiectasis GWAS was carried forward using the rounded effective sample size available from the analysis record.

CRSsNP summary statistics were obtained from GWAS Catalog accession GCST90652532. This GWAS was generated from FinnGen and UK Biobank European participants and included 15,448 cases and 685,602 controls [4]. The phenotype was retained as chronic rhinosinusitis without nasal polyps to distinguish it from nasal polyposis-related chronic rhinosinusitis. In the present study, CRSsNP was included to represent upper airway inflammatory disease within the unified airway disease framework.

Supplementary Methods 2. GWAS summary statistic harmonization

All GWAS summary statistics were reformatted into a standardized structure before downstream analysis. The harmonized format included chromosome, base-pair position, SNP identifier, effect allele, non-effect allele, effect allele frequency, effect estimate, standard error, association *P* value and sample size. Column names were standardized across datasets to ensure compatibility with

downstream LDSC, LAVA, Genomic SEM, PoPS, MAGMA, MTWAS and Mendelian randomization workflows.

Variants were excluded if they had missing SNP identifiers, missing effect or non-effect alleles, missing effect estimates, missing standard errors or invalid *P* values. Duplicate SNPs were removed where necessary. When duplicate entries were present, the variant record with the strongest association evidence was retained. Alleles were checked to ensure interpretable effect directions, and all summary statistics were harmonized so that positive beta values represented increased genetic liability to the corresponding airway disease.

For binary traits with available case-control information, effective sample size was calculated as:

$N\text{eff} = 4 \times N\text{case} \times N\text{control} / (N\text{case} + N\text{control})$.

The effective sample sizes used in downstream analyses were 64,095 for COPD, 444,537 for asthma, 11,477 for bronchiectasis and 60,430 for CRSsNP. Variants in the extended major histocompatibility complex region were excluded from selected downstream visualization and post-GWAS interpretation because of the complex linkage disequilibrium structure in this region.

Supplementary Methods 3. LDSC-based SNP heritability and global genetic correlation

Univariate Linkage Disequilibrium Score Regression (LDSC) was used to estimate observed-scale SNP-based heritability, genomic control inflation, mean χ^2 statistic and LDSC intercept for each airway disease GWAS [5]. LDSC intercepts were used to evaluate whether inflation in GWAS association statistics was likely to reflect polygenic signal rather than uncontrolled population stratification or cryptic relatedness. Mean χ^2 statistics and λGC were interpreted together with LDSC intercepts to characterize the overall genetic architecture of each input GWAS.

Bivariate LDSC was then performed for all six pairwise combinations among COPD, asthma, bronchiectasis and CRSsNP. This analysis estimated pairwise genetic covariance and genetic correlation between airway disease traits. Genetic covariance represents the shared additive genetic component between two traits, whereas genetic correlation represents the standardized proportion of shared genetic architecture after accounting for trait-specific SNP heritability. Positive genetic correlations were interpreted as evidence of concordant shared genetic liability between airway diseases.

Multiple testing across the six pairwise genetic correlation tests was controlled using Bonferroni correction. The LDSC heritability and pairwise genetic correlation results are summarized in Supplementary Tables S1 and S2.

Supplementary Methods 4. Local genetic correlation analysis using LAVA

To evaluate whether shared genetic architecture between airway diseases was concentrated in specific genomic regions, local genetic correlation analysis was performed using LAVA [6].

Unlike global LDSC genetic correlation, which provides a genome-wide average estimate, LAVA estimates local genetic correlations across semi-independent genomic loci.

For each airway disease pair, local heritability was first estimated within each locus. Bivariate local genetic correlation was then calculated for loci with sufficient local genetic signal. The resulting local genetic correlation coefficient quantified the direction and magnitude of shared genetic effects between two diseases within a specific genomic region. Loci with positive local correlations suggested concordant regional genetic liability, whereas negative correlations suggested divergent regional genetic effects.

Local genetic correlation P values were corrected using the Benjamini–Hochberg false discovery rate procedure across tested loci. Significant local genetic correlation signals were visualized using locus-level volcano plots, with the x axis representing local genetic correlation and the y axis representing $-\log10(P)$. This analysis was used to complement global LDSC results and to determine whether shared airway disease genetics showed regional heterogeneity.

Supplementary Methods 5. Assessment of factorability before Genomic SEM

Before fitting the Genomic SEM model, we evaluated whether the genetic covariance matrix of the four airway diseases was suitable for latent-factor modelling. Three complementary analyses were performed: Kaiser–Meyer–Olkin (KMO) assessment, parallel analysis and exploratory factor analysis.

The overall KMO value was 0.808, indicating that the genetic covariance structure was suitable for factor analysis. Trait-specific KMO values were 0.799 for COPD, 0.816 for asthma, 0.788 for bronchiectasis and 0.838 for CRSsNP. Parallel analysis suggested extraction of a single component, supporting a one-factor representation of shared genetic liability across the four airway diseases.

Exploratory factor analysis was performed using a one-factor model with promax rotation. The single factor explained 62.6% of the variance, with SS loading of 2.505. Factor loadings were positive for all four traits: 0.802 for COPD, 0.808 for asthma, 0.850 for bronchiectasis and 0.696 for CRSsNP. Uniqueness values were 0.356 for COPD, 0.347 for asthma, 0.277 for bronchiectasis and 0.515 for CRSsNP, indicating that the shared factor captured a substantial component of the genetic variance across the airway disease traits.

Supplementary Methods 6. Genomic SEM and construction of gAirwayDisease

A common-factor Genomic Structural Equation Modelling model was fitted using the GenomicSEM framework [7]. Multivariable LDSC was first used to estimate the genetic covariance matrix and sampling covariance matrix across COPD, asthma, bronchiectasis and CRSsNP. These matrices were then used as input for the Genomic SEM common-factor model.

A one-factor model was specified in which COPD, asthma, bronchiectasis and CRSsNP loaded onto a single latent genetic factor. This factor was termed gAirwayDisease and was interpreted as

the shared genetic liability underlying major airway diseases. Standardized factor loadings were 0.809 for COPD, 0.836 for asthma, 0.826 for bronchiectasis and 0.658 for CRSsNP. All four loadings were positive and statistically significant, indicating that each airway disease contributed to the shared genetic factor.

Model fit was evaluated using χ^2, degrees of freedom, comparative fit index and standardized root mean square residual. The one-factor model demonstrated excellent fit, with $\chi^2 = 1.152$, df = 2, $P = 0.562$, AIC = 17.152, CFI = 1.000 and SRMR = 0.032. These results supported the use of a single latent factor to summarize shared genetic liability across the four airway diseases.

A genome-wide association analysis of gAirwayDisease was subsequently performed within the Genomic SEM framework. Genome-wide significance was defined as $P < 5 \times 10^{-8}$. The resulting gAirwayDisease GWAS summary statistics were used for downstream spatial mapping, gene prioritization, transcriptome-wide association analysis, regulon analysis and Mendelian randomization.

Supplementary Methods 7. Manhattan plot visualization of gAirwayDisease GWAS

Genome-wide association results for gAirwayDisease were visualized using Manhattan plots. The input file contained chromosome, base-pair position, SNP identifier, effect allele, non-effect allele, effect allele frequency, beta, standard error, association *P* value and sample size. The extended MHC region was removed before visualization to avoid distortion caused by complex linkage disequilibrium in this region.

Manhattan plots were generated using CMplot. Suggestive and genome-wide significance thresholds were displayed at $P < 1 \times 10^{-5}$ and $P < 5 \times 10^{-8}$, respectively. Signals exceeding the genome-wide significance threshold were visually highlighted. These plots were used to summarize the genome-wide distribution of association signals for the GSEM-derived gAirwayDisease factor.

Supplementary Methods 8. gsMap spatial enrichment analysis

We used genetically informed spatial mapping of cells for complex traits (gsMap; version 1.72.3) to identify spatially resolved cells and anatomical regions associated with gAirwayDisease by integrating the latent-factor GWAS summary statistics with spatial transcriptomic data.[8] Briefly, gsMap uses a graph neural network to identify homogeneous spots based on their gene-expression profiles and spatial proximity. Gene specificity scores were calculated for each focal spot by aggregating expression information across neighboring homogeneous spots and were assigned to single-nucleotide polymorphisms within 50 kb of the corresponding gene regions. Each spot-specific SNP annotation was then tested for enrichment of gAirwayDisease heritability using stratified linkage disequilibrium score regression conditional on the baseline annotations. The Cauchy combination test was used to aggregate spot-level P values within each annotated spatial region. The official European linkage disequilibrium reference panel provided by the gsMap workflow was used. Spatial transcriptomic data were obtained from the E16.5_E1S1 coronal section of the Mouse Organogenesis Spatiotemporal Transcriptomic Atlas, comprising 81,125 spatially resolved spots annotated into 25 anatomical regions.

Before analysis, GWAS variants with missing SNP identifiers or invalid association statistics, duplicated variants, and variants absent from the linkage disequilibrium reference panel were excluded.

Supplementary Methods 9. PoPS gene prioritization

Gene prioritization was performed using Polygenic Priority Score (PoPS) version 0.2 [9]. MAGMA gene-level association outputs (.genes.out and .genes.raw) were used as the genetic association input. The analysis used European-ancestry linkage disequilibrium reference data, Ensembl-based protein-coding gene annotations, 116 chunks of the full PoPS feature matrix, and the corresponding control-feature file. PoPS was run using the recommended regression settings. Genes lacking a valid Ensembl identifier, MAGMA association statistic, or required feature information were excluded, and duplicated gene identifiers were removed. Genes were ranked across the complete set of successfully evaluated genes without applying a significance threshold to the PoPS scores.

After PoPS scoring, Ensembl gene identifiers were mapped to gene symbols where possible. Genes were ranked in descending order of PoPS score. Genome-wide ranks and percentiles were calculated across all tested genes. Top genes were visualized using genome-wide prioritization plots and lollipop-style plots. Genes in the highest-ranking percentiles were interpreted as candidate effector genes with polygenic support for shared airway disease liability.

Supplementary Methods 10. MAGMA gene-set enrichment analysis

MAGMA version 1.10, implemented in the FUMA SNP2GENE platform, was used to perform gene-level and competitive gene-set analyses [10]. SNPs were assigned to protein-coding genes using Ensembl v110 gene coordinates on the GRCh37 genome build, without additional upstream or downstream extension. Linkage disequilibrium between variants was estimated using the European subset of the 1000 Genomes Project Phase 3 reference panel. Variants absent from the reference panel and genes without mapped variants or valid association statistics were excluded. Gene-level associations were calculated using the SNP-wise mean model. Competitive gene-set analysis was subsequently performed using 17,023 gene sets from MSigDB v2023.1Hs, including curated gene sets and Gene Ontology biological process, cellular component, and molecular function collections. No additional gene-set size filtering was applied. Multiple testing across all evaluated gene sets was controlled using Bonferroni correction, and adjusted $P < 0.05$ was considered statistically significant.

Supplementary Methods 11. GTEx v8 lung MTWAS analysis

Multi-tissue transcriptome-wide association study (MTWAS) was used to identify associations between genetically predicted gene expression and gAirwayDisease [11]. For the primary tissue-level analysis, we used UK Biobank-compatible GTEx v8 MTWAS expression prediction weights [11,12]. These weights were trained from GTEx v8 tissue-specific expression models, with eQTL SNPs restricted to variants available in UK Biobank GWAS datasets to improve compatibility with UK Biobank-derived phenotypes.

The GSEM-derived gAirwayDisease GWAS summary statistics were used as the trait input. MTWAS tested whether genetically predicted gene expression was associated with gAirwayDisease across GTEx v8 tissues. For the main lung-focused analysis, we extracted MTWAS results from GTEx v8 lung tissue. MTWAS *Z* statistics were used to represent the direction and magnitude of genetically predicted expression associations. Positive MTWAS *Z* values indicated that higher genetically predicted lung expression was associated with increased gAirwayDisease liability, whereas negative values indicated the opposite direction.

For the main table, the top positively associated lung MTWAS genes were selected to highlight genes whose increased genetically predicted lung expression was associated with higher shared airway disease liability. Nominal MTWAS *P* values, prediction model performance metrics, Bonferroni-adjusted *P* values and FDR-adjusted *P* values were retained. Prediction *P* values reported as zero in the original output were displayed as $<2.2 \times 10^{-16}$ to avoid implying an exact zero probability.

Supplementary Methods 12. OneK1K immune-cell MTWAS analysis

To investigate immune-cell-specific genetically regulated expression, MTWAS was performed using OneK1K immune-cell-specific expression prediction weights [11,13]. OneK1K provides single-cell eQTL information from peripheral blood mononuclear cells and enables cell-type-specific mapping of genetic effects on gene expression across major immune cell populations.

The gAirwayDisease GWAS summary statistics were used as input, and MTWAS was conducted across available OneK1K immune cell types. These included T-cell, B-cell, NK-cell, monocyte, dendritic-cell and plasma-cell-related immune contexts. For each gene-cell-type pair, MTWAS estimated a standardized association statistic and corresponding association *P* value. Prediction model performance was summarized using prediction r^2 and prediction *P* values.

For the main OneK1K table, the first five unique genes from the OneK1K MTWAS result table were retained, and all corresponding immune-cell-specific associations for these genes were shown. This approach preserved repeated cell-type-specific associations for genes appearing across multiple immune contexts, allowing interpretation of whether shared airway disease liability converged on specific immune-cell lineages.

Supplementary Methods 13. DICE immune-cell MTWAS sensitivity analysis

As a sensitivity analysis, immune-cell MTWAS was repeated using DICE-based expression prediction weights [11,14]. DICE provides immune-cell and activation-state-specific expression quantitative trait locus resources, enabling evaluation of genetically regulated expression in sorted immune cell populations and stimulated immune contexts.

DICE-based MTWAS was performed using the same gAirwayDisease GWAS summary statistics as input. The analysis tested associations between genetically predicted expression and gAirwayDisease across DICE immune cell types and activation states, including naive and stimulated T-cell contexts, monocyte subsets, B cells, T helper subsets and regulatory T-cell

contexts. The DICE analysis was used to assess whether immune-cell-specific expression associations observed in OneK1K were supported by an independent immune-cell eQTL resource.

DICE results were interpreted as sensitivity evidence rather than as the primary immune-cell MTWAS result, because OneK1K provided a single-cell immune eQTL framework whereas DICE provided sorted immune-cell and activation-state eQTL information.

Supplementary Methods 14. scMORE regulon enrichment analysis

scMORE version 2.0.0 was used to identify immune-cell-specific transcription factor eRegulons associated with gAirwayDisease [15]. The analysis used the preprocessed peripheral-blood single-cell multiome reference provided by the scMORE framework. The single-cell data contained matched gene-expression and chromatin-accessibility information and were represented on the GRCh38 genome assembly. GWAS variant coordinates were harmonized to GRCh38 before SNP-to-peak mapping. Variants with missing chromosome, position, effect estimate, standard error, allele frequency, sample size, or SNP identifier were excluded, as were duplicated variants and variants that could not be uniquely mapped to GRCh38. MAGMA gene-level association statistics for gAirwayDisease were used to quantify gene-level genetic relevance.

scMORE first constructed a global transcription factor–gene regulatory network using a generalized linear model and linked regulatory peaks to target genes using the Signac-based peak-to-gene method. Cell-type specificity was estimated using the modified cosine-similarity method. The analysis was performed using the following parameters: n_targets = 5, perm_n = 1000, theta = 0.5, alpha = 1, buffer = 500, top_n = 5, peak2gene_method = "Signac", infer_method = "glm", method = "cosine", and nSeed = 1234. For each regulon–cell-type pair, scMORE calculated a cell-type specificity score (CTS), genomic risk score (GRS), and trait-relevant regulon score (TRS). Empirical P values were estimated using 1,000 randomly generated control regulons matched for regulon size. Significant regulon–cell-type pairs were required to meet all three prespecified criteria: CTS $P < 0.05$, GRS $P < 0.05$, and TRS $P < 0.05$. All tested regulon–cell-type pairs were retained for quality assessment, and the 36 pairs satisfying all three criteria are reported in Supplementary Table S9.

Supplementary Methods 15. Phenome-wide Mendelian randomization

Phenome-wide two-sample Mendelian randomization was performed to evaluate whether genetically predicted gAirwayDisease was associated with a broad range of downstream clinical and immune-related traits. gAirwayDisease was treated as the genetic exposure. Outcome GWAS datasets were obtained through MR-Base resources and related GWAS datasets accessible through the TwoSampleMR-compatible workflow [16].

Before MR analysis, exposure and outcome datasets were harmonized to align effect alleles. Palindromic SNPs with ambiguous allele frequency information were removed where necessary. Instruments were retained according to the available gAirwayDisease genetic instrument set. Wald ratio estimates were used when only one SNP instrument was available for a given exposure-

outcome pair, whereas inverse-variance weighted estimates were used when multiple SNP instruments were available.

The MR analysis was run using Bonferroni correction for multiple testing. Although the adjusted *P* value column in the output was labelled fdr, it represented the Bonferroni-adjusted *P* value because the MR workflow was executed using Bonferroni correction. The full MR-PheWAS output was retained in Supplementary Table S10. For the main visualization, we focused on more robust associations supported by more than five SNP instruments and Bonferroni-adjusted $P < 0.05$. This filtering step was used to reduce overinterpretation of single-SNP Wald ratio signals and to emphasize more stable multi-instrument MR associations.

MR results were interpreted as phenome-wide genetic evidence linking gAirwayDisease liability to downstream traits, rather than as definitive proof of causal mechanisms. Associations with respiratory, allergic and immune-related outcomes were considered particularly relevant to the biological interpretation of shared airway disease liability.

Supplementary Methods 16. Software and statistical reporting

All analyses were performed using R and established GWAS summary-statistic analysis tools. LDSC was used for SNP heritability and global genetic correlation estimation. LAVA was used for local genetic correlation analysis. Genomic SEM was used to construct the gAirwayDisease latent factor and perform latent-factor GWAS. gsMap, PoPS and MAGMA were used for spatial mapping, gene prioritization and pathway enrichment, respectively. MTWAS was used for lung tissue and immune-cell genetically regulated expression analyses. scMORE was used for immune-cell-specific regulon enrichment analysis. TwoSampleMR-compatible workflows were used for phenome-wide Mendelian randomization.

Unless otherwise specified, two-sided *P* values were reported. Genome-wide significance was defined as $P < 5 \times 10^{-8}$ for GWAS analyses. Bonferroni correction was applied where a fixed number of tests was prespecified, including pairwise genetic correlations and phenome-wide MR screening. FDR correction was used for local genetic correlation and enrichment-style analyses where appropriate. For tabular reporting, very small *P* values displayed as zero in software output were reported as $<2.2 \times 10^{-16}$ when applicable.

References

1. Newbury A, Elhussein A, Gürsoy G. Multi-domain rule-based phenotyping algorithms enable improved GWAS signal. *NPJ Digit Med*. 2025;8:499. doi:10.1038/s41746-025-01815-8.
2. Zhou W, Kanai M, Wu K-HH, Rasheed H, Tsuo K, Hirbo JB, et al. Global Biobank Meta-analysis Initiative: powering genetic discovery across human disease. *Cell Genomics*. 2022;2(10):100192. doi:10.1016/j.xgen.2022.100192.

3. Sakaue S, Kanai M, Tanigawa Y, Karjalainen J, Kurki M, Koshiba S, et al. A cross-population atlas of genetic associations for 220 human phenotypes. *Nat Genet*. 2021;53(10):1415–1424. doi:10.1038/s41588-021-00931-x.
4. Saarentaus EC, Fischer-Rasmussen K, Sliz E, Bizaki-Vallaskangas A, Laitinen T, Toppila-Salmi S, et al. 131 genetic loci highlight immunological pathways and tissues in nasal polyposis and asthma. *Nat Commun*. 2025;16:9879. doi:10.1038/s41467-025-64847-4.
5. Bulik-Sullivan BK, Loh PR, Finucane HK, Ripke S, Yang J, Patterson N, et al. LD Score regression distinguishes confounding from polygenicity in genome-wide association studies. *Nat Genet*. 2015;47(3):291–295. doi:10.1038/ng.3211.
6. Werme J, van der Sluis S, Posthuma D, de Leeuw CA. An integrated framework for local genetic correlation analysis. *Nat Genet*. 2022;54(3):274–282. doi:10.1038/s41588-022-01017-y.
7. Grotzinger AD, Rhemtulla M, de Vlaming R, Ritchie SJ, Mallard TT, Hill WD, et al. Genomic structural equation modelling provides insights into the multivariate genetic architecture of complex traits. *Nat Hum Behav*. 2019;3(5):513–525. doi:10.1038/s41562-019-0566-x.
8. Song L, Chen W, Hou J, Guo M, Yang J. Spatially resolved mapping of cells associated with human complex traits. *Nature*. 2025;641:932–941. doi:10.1038/s41586-025-08757-x.
9. Weeks EM, Ulirsch JC, Cheng NY, Trippe BL, Fine RS, Miao J, et al. Leveraging polygenic enrichments of gene features to predict genes underlying complex traits and diseases. *Nat Genet*. 2023;55(8):1267–1276. doi:10.1038/s41588-023-01443-6.
10. de Leeuw CA, Mooij JM, Heskes T, Posthuma D. MAGMA: generalized gene-set analysis of GWAS data. *PLoS Comput Biol*. 2015;11(4):e1004219. doi:10.1371/journal.pcbi.1004219.
11. Song S, Wang L, Hou L, Liu JS. Partitioning and aggregating cross-tissue and tissue-specific genetic effects to identify gene-trait associations. *Nat Commun*. 2024;15:5769. doi:10.1038/s41467-024-49924-4.
12. GTEx Consortium. The GTEx Consortium atlas of genetic regulatory effects across human tissues. *Science*. 2020;369(6509):1318–1330. doi:10.1126/science.aaz1776.
13. Yazar S, Alquicira-Hernandez J, Wing K, Senabouth A, Gordon MG, Andersen S, et al. Single-cell eQTL mapping identifies cell type-specific genetic control of autoimmune disease. *Science*. 2022;376(6589):eabf3041. doi:10.1126/science.abf3041.
14. Schmiedel BJ, Singh D, Madrigal A, Valdovino-Gonzalez AG, White BM, Zapardiel-Gonzalo J, et al. Impact of genetic polymorphisms on human immune cell gene expression. *Cell*. 2018;175(6):1701–1715.e16. doi:10.1016/j.cell.2018.10.022.
15. Ma Y, Yao Y, Zhou Y, Dai W, Li J, Gui Y, et al. Integrating polygenic signals and single-cell multiomics identifies cell-type-specific regulomes critical for immune- and aging-related diseases. *Nat Aging*. 2025. doi:10.1038/s43587-025-01027-5.
16. Hemani G, Zheng J, Elsworth B, Wade KH, Haberland V, Baird D, Laurin C, Burgess S, Bowden J, Langdon R, et al. The MR-Base platform supports systematic causal inference across the human phenome. *eLife*. 2018;7:e34408. doi:10.7554/eLife.34408.

Supplementary Results

Supplementary Results 1. GWAS architecture and LDSC quality metrics

The four harmonized GWAS datasets included COPD, asthma, bronchiectasis and CRSsNP. COPD had 16,712 cases and 389,099 controls, corresponding to an effective sample size of 64,095. Asthma had 121,940 cases and 1,254,131 controls, corresponding to an effective sample size of 444,537. CRSsNP had 15,448 cases and 685,602 controls, corresponding to an effective sample size of 60,430. Bronchiectasis was analysed using the rounded effective sample size available from the analysis record, with Neff = 11,477. Detailed data sources, harmonized phenotype labels, sample sizes and LDSC statistics are provided in Supplementary Table S1.

Univariate LDSC showed observed-scale SNP heritability estimates of 0.1686 for COPD, 0.0770 for asthma, 0.1038 for bronchiectasis and 0.0887 for CRSsNP. LDSC intercepts were 1.0065 for COPD, 1.0826 for asthma, 0.9950 for bronchiectasis and 1.0359 for CRSsNP. Mean χ^2 values were 1.2219 for COPD, 1.7614 for asthma, 1.0188 for bronchiectasis and 1.1419 for CRSsNP. The corresponding λGC values were 1.1973, 1.4890, 1.0195 and 1.1270, respectively.

Supplementary Results 2. Pairwise genetic covariance and genetic correlation

All six pairwise LDSC genetic correlations were positive. COPD–asthma showed a genetic covariance of 0.0709 and genetic correlation of 0.685. COPD–bronchiectasis showed a genetic covariance of 0.0653 and genetic correlation of 0.677. COPD–CRSsNP showed a genetic covariance of 0.0581 and genetic correlation of 0.508. Asthma–bronchiectasis showed a genetic covariance of 0.0412 and genetic correlation of 0.666. Asthma–CRSsNP showed a genetic covariance of 0.0404 and genetic correlation of 0.552. Bronchiectasis–CRSsNP showed a genetic covariance of 0.0394 and genetic correlation of 0.638.

The corresponding genetic correlation standard errors ranged from approximately 0.048 to 0.169. All pairwise correlations were statistically significant, with P values ranging from 1.01×10^{-46} for COPD–asthma to 4.59×10^{-2} for asthma–CRSsNP. Full covariance, correlation, standard error and P-value estimates are provided in Supplementary Table S2.

Supplementary Results 3. Local genetic correlation patterns

LAVA was used to estimate local genetic correlations across semi-independent genomic loci for each disease pair. The COPD–asthma comparison showed local correlation signals with positive regional estimates. COPD–bronchiectasis and asthma–bronchiectasis showed positive local genetic sharing at selected loci, with labelled loci representing the strongest signals in each panel. COPD–CRSsNP, asthma–CRSsNP and bronchiectasis–CRSsNP also showed multiple local genetic correlation signals, including loci exceeding the FDR-defined threshold.

The strongest local signals were visualized as labelled loci in each disease-pair volcano plot. Across panels, the significant local signals were predominantly positive, consistent with the positive genome-wide genetic correlations observed by LDSC.

Supplementary Results 4. Factorability assessment and exploratory factor analysis

The genetic covariance matrix showed adequate suitability for latent-factor modelling. The overall KMO value was 0.808. Trait-specific KMO values were 0.799 for COPD, 0.816 for asthma, 0.788 for bronchiectasis and 0.838 for CRSsNP. Parallel analysis supported extraction of a single common component.

Exploratory factor analysis using a one-factor model showed positive loadings for all four airway diseases. COPD loaded 0.802 onto the factor, asthma loaded 0.808, bronchiectasis loaded 0.850 and CRSsNP loaded 0.696. The single factor had SS loading of 2.505 and explained 62.6% of the variance. Uniqueness values were 0.356 for COPD, 0.347 for asthma, 0.277 for bronchiectasis and 0.515 for CRSsNP. These results are provided in Supplementary Table S3.

Supplementary Results 5. Confirmatory Genomic SEM model

The confirmatory one-factor Genomic SEM model defined the latent factor gAirwayDisease. In the model, COPD, asthma, bronchiectasis and CRSsNP were specified as observed disease traits loading onto the common factor. Standardized factor loadings were 0.809 for COPD, 0.836 for asthma, 0.826 for bronchiectasis and 0.658 for CRSsNP. The corresponding unstandardized estimates were 0.343 for COPD, 0.228 for asthma, 0.188 for bronchiectasis and 0.177 for CRSsNP.

Residual variance estimates were 0.062 for COPD, 0.022 for asthma, 0.016 for bronchiectasis and 0.041 for CRSsNP. Standardized residual variances were 0.346, 0.300, 0.318 and 0.567, respectively. The model fit statistics were $\chi^2 = 1.152$, df = 2, P = 0.562, AIC = 17.152, CFI = 1.000 and SRMR = 0.032. Full model parameters are shown in Supplementary Table S4.

Supplementary Results 6. Spatial mapping and PoPS prioritization

gsMap analysis showed spatially heterogeneous enrichment of gAirwayDisease-associated genetic signals across anatomical domains. Enrichment signals were visualized on a sagittal anatomical reference section. The strongest visible signals were concentrated in airway- and epithelial-associated regions, with additional annotated domains including lung, cartilage, connective tissue, muscle and other tissue compartments.

PoPS analysis ranked genes genome-wide by priority score. The top 1% tier included 202 genes, the top 5% tier included 809 genes and the top 10% tier included 1,011 genes, compared with 18,206 genes assigned to the background set. The top ten PoPS-prioritized genes were SMAD3, GATA3, ESR1, STAT5A, UBC, IL1R1, RUNX3, STAT5B, APOE and STAT6. Their scores were 3.652, 3.046, 2.528, 2.523, 2.483, 2.415, 2.339, 2.268, 2.251 and 2.203, respectively.

Supplementary Results 7. MAGMA gene-set enrichment

MAGMA gene-set enrichment analysis identified multiple pathways enriched for gAirwayDisease-associated genetic signals. The enriched categories were dominated by immune activation and transcriptional regulation. Top terms included B-cell activation, alpha-beta T-cell activation, CD4-positive T-cell activation, regulation of T-cell proliferation, positive regulation of macromolecule biosynthetic process, positive regulation of biosynthetic process, regulation of RNA metabolic process and RUNX3/BCL11B-related T-cell lineage transition.

The strongest enriched term reached approximately −log10 adjusted P ≈ 6.7. Several gene sets exceeded the adjusted significance threshold, as indicated by the dashed line in the enrichment bar plot.

Supplementary Results 8. GTEx v8 MTWAS across tissues

GTEx v8-based MTWAS was performed across human tissues to identify genetically predicted expression associated with gAirwayDisease. The complete cross-tissue MTWAS results are provided in Supplementary Table S5. The UK Biobank-compatible GTEx v8 MTWAS output is provided in Supplementary Table S6.

In GTEx v8 lung tissue, the top ten positively associated genes were SLC9A2, ORMDL3, SIPA1, RAPGEFL1, GNGT2, INTS12, CDK2AP1, EEFSEC, ZSWIM7 and HR. SLC9A2 showed the strongest association, with MTWAS Z = 6.955, MWAS P = 3.513×10^{-12}, predicted R^2 = 0.0679 and TWAS Bonferroni P = 4.110×10^{-9}. ORMDL3 showed MTWAS Z = 5.825, MWAS P = 5.704×10^{-9} and TWAS Bonferroni P = 6.286×10^{-6}. RAPGEFL1 had the highest prediction model R^2 among the top ten lung signals, with predicted R^2 = 0.215.

All top ten lung signals showed positive MTWAS Z values, indicating that higher genetically predicted lung expression was associated with increased gAirwayDisease liability in these selected gene-tissue pairs.

Supplementary Results 9. OneK1K and DICE immune-cell MTWAS

OneK1K immune-cell MTWAS identified recurrent immune-cell-specific expression signals associated with gAirwayDisease. IL18R1 was the strongest recurrent signal and appeared across CD8 S100B, CD8 ET, CD4 NC and CD8 NC cells. The strongest IL18R1 association was observed in CD8 S100B cells, with MTWAS Z = 9.962 and MWAS P = 2.226×10^{-23}. Additional IL18R1 signals were observed in CD8 ET cells (Z = 9.826), CD4 NC cells (Z = 9.033) and CD8 NC cells (Z = 8.722).

Other top OneK1K genes included PSMD3, ORMDL3, HMG20A and CAMK4. PSMD3 was associated in CD4 NC cells with Z = 7.376. ORMDL3 was associated in both CD4 NC and CD8 NC cells, with Z = 5.825. HMG20A was associated in CD4 ET cells with Z = 5.735. CAMK4 appeared across CD4 ET, CD4 NC and B MEM cells, each with Z = 5.497. The complete OneK1K results are provided in Supplementary Table S7.

DICE-based immune-cell MTWAS was performed as an independent immune-cell eQTL sensitivity analysis. The complete DICE immune-cell MTWAS results are provided in Supplementary Table S8.

Supplementary Results 10. scMORE regulon enrichment

scMORE identified 36 significant regulon-cell type pairs using the combined criteria of CTS $P < 0.05$, GRS $P < 0.05$ and TRS $P < 0.05$. Significant regulons were detected across pDCs, mDCs, B cells, CD14+ monocytes, FCGR3A+ monocytes, CD4+ T cells, CD8+ T cells and NK cells. The complete scMORE output is provided in Supplementary Table S9.

In pDCs, significant regulons included BCL11A, SPIB, TCF4 and ZNF521. SPIB had CTS = 3.521, GRS = 3.781 and TRS = 3.757. In B cells, eight regulons were significant: BCL11A, EBF1, IKZF3, JAZF1, POU2F2, SPIB, TCF4 and TRERF1. BCL11A had the highest B-cell TRS value, with CTS = 3.805, GRS = 4.168 and TRS = 3.922.

In monocytes, IKZF1 and KLF4 were significant in both CD14+ and FCGR3A+ subsets. KLF4 showed GRS values of 4.322 in CD14+ monocytes and 4.463 in FCGR3A+ monocytes. In CD4+ T cells, significant regulons included ETS1, FOXO1, HIVEP2, IKZF1, RUNX1 and STAT4. In CD8+ T cells, significant regulons included ETS1, FOXO1, HIVEP2, IKZF1, NFATC3, RUNX1 and STAT4. RUNX1 had the highest TRS among CD8+ T-cell regulons, with TRS = 3.979. In NK cells, significant regulons included ETS1, FOXO1, GTF3A, HIVEP2, RUNX1 and STAT4.

Supplementary Results 11. Phenome-wide Mendelian randomization

Phenome-wide Mendelian randomization was performed using genetically predicted gAirwayDisease as the exposure. The complete MR-PheWAS output is provided in Supplementary Table S10. The main figure retained outcomes with more than five instrumental SNPs and Bonferroni-adjusted $P < 0.05$.

Significant associations were observed across respiratory, allergic, lung function and immune-related traits. Positive associations included asthma-related outcomes, allergic rhinitis-related outcomes and air pollution-related asthma outcomes. Negative associations included lung function traits such as FEV1- and FVC-related measures. In the volcano plot, the strongest associations reached $-\log10$ Bonferroni-adjusted P values greater than 100. Point size represented the number of instrumental SNPs, and both positive and negative MR effect estimates were observed after filtering for multi-SNP-supported associations.
