# Supplementary material for: Genetic Evidence for Unified Airway Disease: Shared Epithelial and Immune Architecture Across Major Airway Diseases
Source: Int J Mol Sci. 2026 Aug 20;27(16):7450. doi: 10.3390/ijms27167450 (PMC13513354; doi:10.3390/ijms27167450)
Supplement: Supplementary file 1 [file ijms-27-07450-s001.zip › Supplementary Figures.pdf]

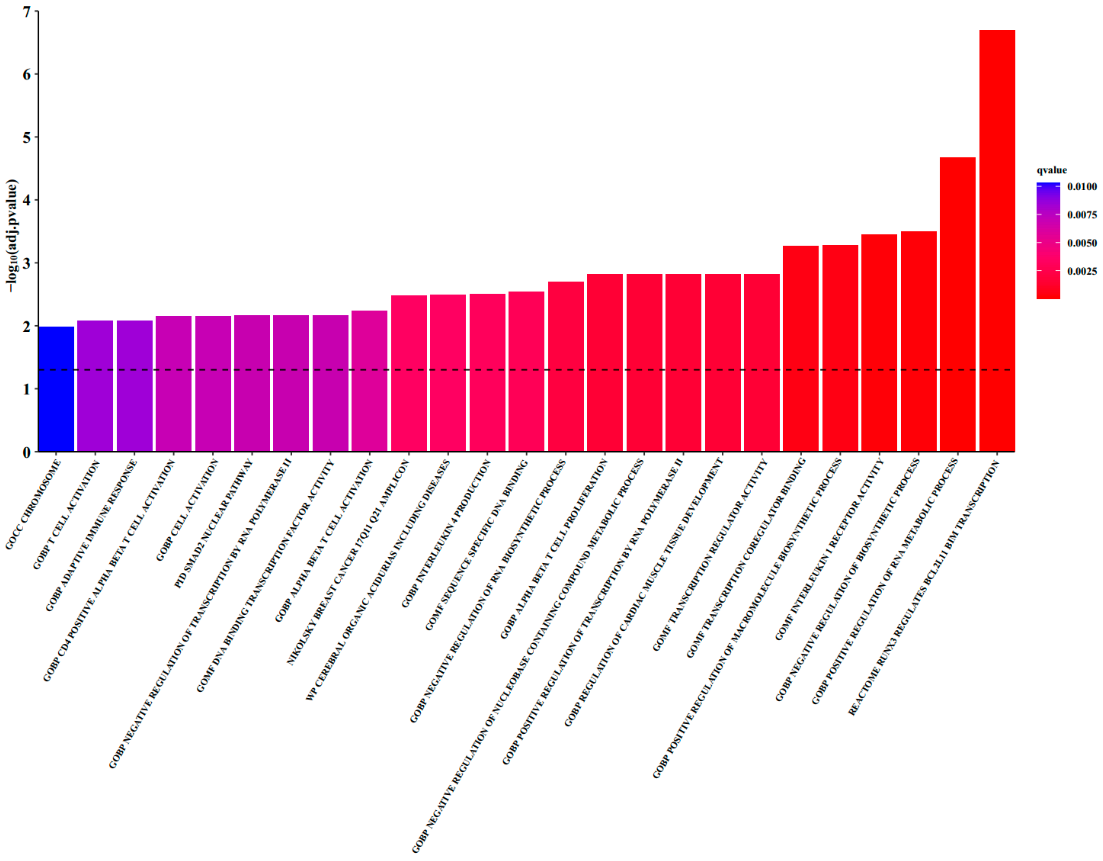

Figure S1. MAGMA gene-set enrichment analysis of gAirwayDisease.

Bar plot shows top MAGMA-enriched gene sets. The y-axis represents −log10 adjusted P value; the dashed line indicates the adjusted significance threshold. Enriched terms were dominated by immune activation and transcriptional regulatory pathways.

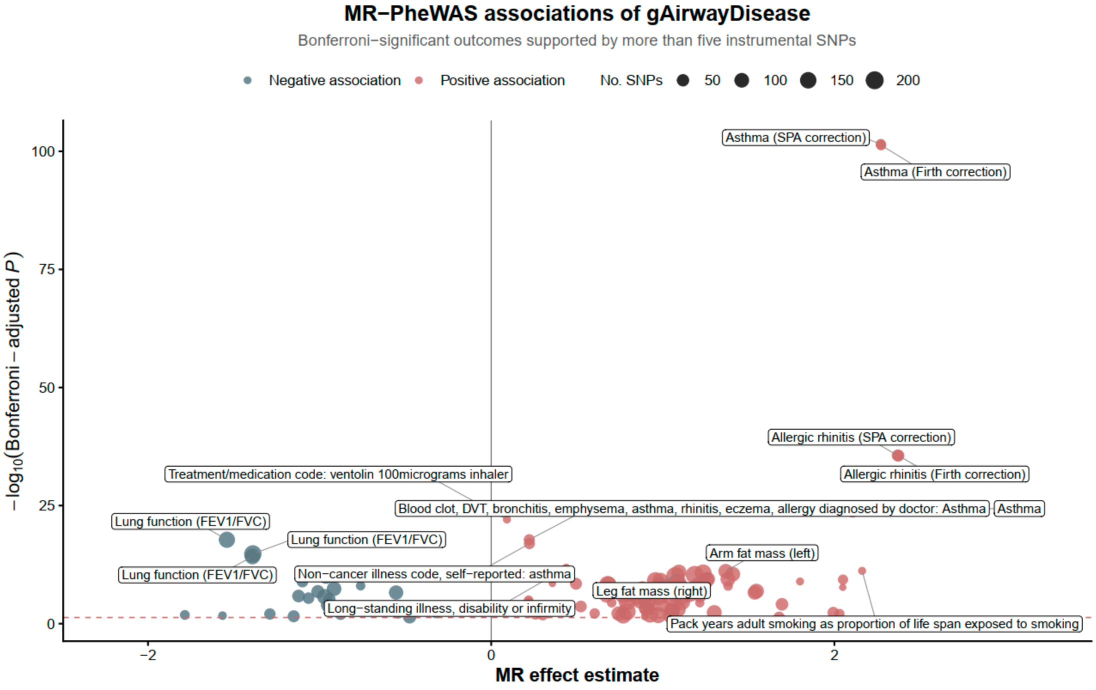

Figure S2. MR-PheWAS associations of genetically predicted gAirwayDisease.

Volcano plot shows phenome-wide MR associations between gAirwayDisease and IEU outcomes. The x-axis shows MR effect estimates; the y-axis shows −log10 Bonferroni-adjusted P value. Significant associations clustered around respiratory, allergic, lung function, and immune-cell traits.
